# Investigation of the genetic effect of 56 tobacco-smoking susceptibility genes on DNA methylation and RNA expression in human brain

**DOI:** 10.3389/fpsyt.2022.924062

**Published:** 2022-08-18

**Authors:** Zhongli Yang, Jiekun Yang, Ying Mao, Ming D. Li

**Affiliations:** ^1^State Key Laboratory for Diagnosis and Treatment of Infectious Diseases, National Clinical Research Center for Infectious Diseases, National Medical Center for Infectious Diseases, Collaborative Innovation Center for Diagnosis and Treatment of Infectious Diseases, The First Affiliated Hospital, Zhejiang University School of Medicine, Hangzhou, China; ^2^Research Center for Air Pollution and Health, Zhejiang University, Hangzhou, China

**Keywords:** brain, eQTL, functional SNPs, gene expression, methylation, smoking

## Abstract

Although various susceptibility genes have been revealed to influence tobacco smoking, the underlying regulatory mechanisms between genetic variants and smoking are poorly understood. In this study, we investigated *cis*-expression quantitative trait loci (*cis*-eQTLs) and methylation quantitative trait loci (mQTLs) for 56 candidate smoking-linked genes using the BrainCloud cohort samples. An eQTL was revealed to significantly affect *EGLN2* expression in the European sample and two mQTLs were respectively detected in CpG sites in *NRXN1* and *CYP2A7*. Interestingly, we found for the first time that the minor allele of the single nucleotide polymorphism (SNP) rs3745277 located in *CYP2A7P1* (downstream of *CYP2B6*) significantly decreased methylation at the CpG site for *CYP2A7* (cg25427638; *P* = 5.31 × 10^–7^), reduced expression of *CYP2B6* (*P* = 0.03), and lowered the percentage of smokers (8.8% vs. 42.3%; Odds Ratio (OR) = 0.14, 95% Confidence Interval (CI): 0.02–0.62; *P* = 4.47 × 10^–3^) in a dominant way for the same cohort sample. Taken together, our findings resulted from analyzing genetic variation, DNA methylation, mRNA expression, and smoking status together using the same participants revealed a regulatory mechanism linking mQTLs to the smoking phenotype. Moreover, we demonstrated the presence of different regulatory effects of low-frequency and common variants on mRNA expression and DNA methylation.

## Introduction

Tobacco smoking is the leading preventable cause of death throughout the world, and one of the three leading components of the global disease burden (1). It causes more than 7 million deaths annually, with about 10% of these resulting from second-hand smoke. Even though most smokers realize that smoking causes many types of cancer and affects a person’s overall health (2), an estimated 1.1 billion people still smoke cigarettes worldwide, and about 80% of these live in low- and middle-income countries (1). Nicotine dependence (ND), with an average heritability of 0.56 (3), is the primary factor maintaining smoking behavior and predicting failures of smoking cessation.

With efforts to identify genetic susceptibility loci for smoking during the past decades, a total of 14 linkage regions and 47 unique loci in 60 susceptibility genes have been revealed (4, 5). Most variants identified within these genetic susceptibility loci are located in non-coding regions, an observation which is in line with findings from other complex traits: i.e., variations in non-coding regulatory sequences contribute to the genetics of complex traits (6). However, we know little about the mechanisms by which most regulatory variants act (7). With the rise of massively parallel sequencing technologies, recent studies have characterized multiple levels of gene regulation, including chromatin states, transcription factor (TF) binding footprints, profiles or different epigenetic marks, and posttranscriptional modifications that enable us to probe the control of regulatory variants for transcriptional processes (7, 8). Both *cis*-expression quantitative trait loci (*cis*-eQTLs) and methylation quantitative trait loci (mQTLs) mapped for these molecular phenotypes have been used to nominate single nucleotide polymorphisms (SNPs), which are then tested for their associations with different types of drug addiction such as alcohol (9), heroin (10), and smoking (11).

Hancock et al. (11) found several SNPs that were associated with both *CHRNA5* methylation and expression in addiction-related brain regions and with the risk of ND across diverse ancestry groups. However, unlike the comparatively well-characterized chromosome 15q25.1 region, eQTLs and mQTLs mapped to other ND candidate genes in human brain tissues have rarely been reported. Further, because of the difficulties of human brain specimen collection, datasets containing regulatory phenotypes in the brain are limited. For a dataset with the information on DNA methylation, mRNA expression, and ND phenotypes collected from the same participants, it becomes even more challenging. Here, for the first time, we intended to link SNPs, DNA methylation, mRNA expression, and smoking status together within a single cohort’s study. Although smoking status is used less commonly than ND-related phenotypes such as smoking quantity or the Fagerström Test for Nicotine Dependence (FTND) score, this phenotypic measure could be used to tag some of the same linkage regions and susceptibility genes as efficiently as other ND-related measures (4, 5).

This study had the following three objectives: (1) to determine *cis*-regulatory loci (eQTLs and mQTLs) for a collection of ND susceptibility genes; (2) to characterize significant *cis*-eQTLs and mQTLs by integrating regulatory features from the Roadmap Epigenomics (12), the Encyclopedia of DNA Elements (ENCODE) (13), and Genotype–Tissue Expression (GTEx) (14) Projects; and (3) to combine *cis*-regulatory variants, DNA methylation, mRNA expression, with smoking status to explore their biological mechanisms.

## Materials and methods

### The BrainCloud cohort’s study samples

We used the BrainCloud^1^ cohort’s study samples to identify genetic variants associated with expression and methylation for 56 ND susceptibility genes (5). The SNP genotypes of each sample were either obtained directly from genotype arrays (Illumina Human1M-Duo and HumanHap650Y) provided by the website or imputed on the basis of the 1000 Genomes Project Phase 3 reference panel. Data on mRNA and DNA methylation were available from the post-mortem prefrontal cortex of human participants who had no neuropathologic or neuropsychiatric diagnoses and no reported alcohol or other drug abuse or positive toxicology results (15, 16). For detailed information on the 56 genes of interest located in 46 genetic susceptibility loci in this study, please refer to Supplementary Table 1. Because the *CHRNA5/A3/B4* gene cluster has been reported by others (11) on the same dataset, we excluded this region from the current study. We obtained the data *via* the database of Genotypes and Phenotypes (dbGaP; Accession Number phs000417.v2.p1) and the BrainCloud project website (see text footnote 1).Gene expression data also are available through the dataset deposited in National Center for Biotechnology Information (NCBI) Gene Expression Omnibus (GEO) with series number GSE30272.

In the original dataset, data on SNP genotypes are available for 270 participants, whereas mRNA expression and DNA methylation data are available only for subsets of 269 and 108 subjects, respectively. To eliminate significant effects of developmental life stages and be consistent with the typical age of onset for smoking, only post-childhood subjects (i.e., older than 10 years) were included in this study, which resulted in 178 [94 African Americans (AAs) and 84 European Americans (EAs)] and 60 (31 AAs and 29 EAs) samples with both expression and methylation data, respectively. Please refer to Table 1 for sample characteristics.

**TABLE 1:** Characteristics of study participants.

Sample	mRNA expression		DNA methylation	
	AA	EA	AA	EA
Sample size: *N*	94	84	31	29
Age (years): mean (*SD*)	39.8 (15.9)	36.8 (18.3)	44.2 (18.9)	43.3 (19.5)
Female: *N* (%)	34 (36.2)	25 (29.8)	15 (48.4)	13 (44.8)
Smoker: *N* (%)	30 (31.9)	16 (19.0)	8 (25.8)	6 (20.7)
PMI (hours): mean (*SD*)	33.3 (15.6)	27.4 (15.1)	33.8 (15.4)	29.0 (15.3)
pH: mean (*SD*)	6.6 (0.3)	6.5 (0.3)	6.6 (0.3)	6.5 (0.3)
RIN: mean (*SD*)	8.1 (0.7)	8.2 (0.8)	8.1 (0.6)	8.0 (1.0)

AA, African American; EA, European American; SD, standard deviation; pH, potential of hydrogen; PMI, post-mortem interval; RIN = RNA integrity number.

### Genome-wide covariate and surrogate variable analysis

We first analyzed the contribution of each of the demographic (age, sex, and race) and technical [post-mortem interval, pH, RNA integrity number (RIN), and batch] covariates to the degree of genome-wide expression and methylation. The Kolmogorov-Smirnov (KS) test was used to capture the differences in the correlation *p*-value distributions for each covariate. Except for covariate effects of demographic variables, we paid special attention to post-mortem quantitative factors such as interval, tissue pH status, RIN, and batch effects. Because of the significant minor allele frequency (MAF) differences observed between the AA and EA samples (4), a robust linear regression model with adjustment for covariates was implemented for each ethnic group separately. We also performed surrogate variable analysis on the obtained residuals across the whole genome using the R package “sva” (17). No surrogate variable was found in either AA or EA samples, which verified the elimination of known and unknown factors after the above covariate adjustment. These residuals, instead of the original raw levels, were used for further data analysis.

### Selection of probes and genotype imputation

For expression data of the 56 genes included in the study, 6, 34, and 17 genes had 0, 1, or > 1 expression probes, respectively. For each of those 17 genes with more than one probe, the specific probe covering all transcripts of the target gene and showing the highest intensity was selected, expect for *COMT* and *DLC1* genes, for which we made our decisions based solely on probe position and intensity, respectively. All the 50 selected expression probes contained no SNP in their sequences (Supplementary Table 2).

For the methylation data, 98 CpG sites were identified and measured for 50 of the 56 genes (Supplementary Table 2). The genotype imputation interval for each gene was assigned according to the widest genomic range determined by its start and end positions and the CpG site position(s), plus 1 Mb on both sides (16, 18). If the intervals for several adjacent genes overlapped, an all-inclusive genomic interval was chosen on the corresponding chromosome for imputation.

Genotype imputation for each genomic interval was conducted with reference to the haplotype panel of the 1000 Genomes Project Phase 3 integrated variant set release^2^ using IMPUTE2 with the default settings (19) (Supplementary Table 3). We used a cutoff of 0.3 for the “info” metric, comparable to the r-squared metrics implemented by other programs such as MaCH and Beagle (20) to remove poorly imputed variations (21, 22). To have the same set of SNPs for both ethnic samples, any loci with a call rate of < 95% and a Hardy-Weinberg equilibrium (HWE) *p*-value of < 0.001 was excluded from the combined sample for either expression or methylation data.

### Association quantitative trait loci analysis and multiple testing correction

Genotype dosage data, including the imputed variants after quality control measures, were analyzed for associations with expression and methylation phenotypes (the residuals described in the preceding section) using PLINK (23). Linear regression analysis was performed to test for correlation between the residuals and the number of minor alleles for each variant under the additive genetic model. From this analysis, an asymptotic *p*-value from the Wald statistic was obtained as a measure of association for each variant with any extent of expression or methylation of a given gene.

To correct for the number of SNPs tested for each phenotype, a region-wide empirical *p*-value was computed for the asymptotic *p*-value for each variation by using 1,000 permutations provided in PLINK (23). To correct for the number of comparisons being investigated for expression or methylation, a false discovery rate (FDR) threshold was calculated on the basis of the region-wide empirical *p*-values by using the *fwer2fdr* function of the R package “multtest.”

### Variant annotation and *post hoc* analysis

For those genetic loci with the number of aggregated QTLs *n* ≥ 10, linkage disequilibrium (LD) blocks were defined following Gabriel et al. (24) in Haploview (Supplementary Figures 1–3). Tracks from the 1000 Genomes Browser^3^ were added to illustrate the relative position of each QTL with respect to its corresponding expression or methylation probes and other regulatory features (i.e., promoter regions and TF binding sites) found in multiple cell lines.

Because the *cis*-regulatory variants in *NRXN1*, *CYP2A7*, and *EGLN2* account for the majority of the total significant QTLs identified, we conducted *post hoc* analysis for these three genes. Pearson correlations between corresponding methylation and expression extents were computed. On the basis of the “smoke at death” and “smoking history” information available for this sample, we classified smoking status for each subject as a smoker if he/she smoked at death with a positive or missing smoking history or did not smoke at death but had a positive smoking history and non-smoker if both his/her smoking measures were negative. A total of 36 subjects with missing smoking history plus missing or negative smoking at death were considered as missing smoking status. Please find specific numbers (percentages) of smokers in Table 1.

Student’s *t*-test was used to compare methylation or expression differences between subjects with distinct race, smoking status, or genotypes. Fisher’s exact test was implemented in the analysis of contingency tables formed by any combination of subjects’ ethnic identity, smoking status, and variant minor allele copies. All the above-mentioned statistical tests were performed in R.

## Results

### *Cis*-methylation quantitative trait loci mapping analysis for 56 susceptibility genes

We found 138 *cis*-mQTLs in 10 genes (*NRXN1*, *PDE1C*, *CHRM2*, *TAS2R38*, *CHRNA2*, *DBH*, *PTEN*, *CHRM1*, *NRXN3*, and *CYP2A7*) with region-wide significance; all of which were ethnicity-specific except for *CYP2A7* (Table 2). Phenotype-wide significant variants accounted for 102 of them. Among the 138 region-wide significant variants, 42 and 68 are for CpG sites in *NRXN1* (cg10917619) and *CYP2A7* (cg25427638), respectively. The *cis*-mQTL for *NRXN1*, with all the associated variants in complete LD, was EA-specific, whereas the one for *CYP2A7* was observed in both the AA and EA samples. Although the *cis*-mQTLs for *CYP2A7* formed more than one haplotype block, the D’ values among them are high (D’ ≥ 0.75 in AAs and ≥ 0.63 in EAs). Corresponding LD plots are shown in Supplementary Figure 1 for *EGLN2* and Supplementary Figure 2 for *NRXN1* in EAs, and Supplementary Figures 3A,B for *CYP2A7* in AAs and EAs, respectively. Furthermore, 56 of the 68 region-wide significant variants in the *CYP2A7* mQTL showed phenotype-wide significance in both ethnic groups.

**TABLE 2:** Significant CpG-SNP pairs with region- or phenotype (pheno)-wide *cis* associations in the AA and EA samples.

Gene	CpG methylation probe ID	RefGene Group	SNP ID (A1/A2)	SNP position	Distance to CpG	AA (*N* = 31)				EA (*N* = 29)			
						A1 Freq	*R* ^2^	Region -wide *P*	Pheno -wide *P*	A1 Freq	*R* ^2^	Region -wide *P*	Pheno -wide *P*
*NRXN1*	cg10917619	5′-UTR	rs7567632 (C/T)	51,198,384	–57,243	0.10	0.13	1	1	0.19	0.51	**0.033**	0.054
			rs6545187 (C/T)	51,200,546	–55,081	0.23	0.23	0.098	1	0.40	0.55	**0.015**	**0.03**
			rs7574611 (A/C)	51,214,993	–40,634	0.16	0.27	0.099	1	0.34	0.70	**0.001**	**0.002**
			rs7594170 (G/A)	51,237,767	–17,860	0.35	0.20	1	1	0.40	0.61	**0.004**	**0.008**
			rs2163018 (T/C)	51,304,238	48,611	0.11	0.14	1	1	0.19	0.51	**0.033**	0.066
*PDE1C*	cg22131691	Body	rs73303752 (G/A)	31,495,561	–615,427	0.11	0.55	**0.038**	0.067	0	NA	1	1
*CHRM2*	cg04748704	TSS1500	rs35259000 (C/CTA)	13,715,061	599,818	0.13	0.54	**0.015**	**0.024**	0.07	0	1	1
			rs1528099 (A/G)	137,167,984	614,741	0.26	0.50	**0.038**	0.07	0.07	0	1	1
*TAS2R38*	cg25481253	1*^st^* Exon	rs6949261 (G/C)	141,980,921	307,537	0.42	0.46	**0.05**	0.07	0.09	0.03	1	1
	cg03017475		rs1110074 (T/C)	141,210,054	–464,287	0.24	0	1	1	0.47	0.46	**0.019**	**0.031**
			rs4726505 (A/C)	141,949,743	275,402	0.41	0.50	**0.017**	**0.032**	0.09	0	1	1
*CHRNA2*	cg02953306	5′-UTR	rs78908411 (T/A)	27,331,607	–5,019	0.07	0.55	**0.009**	**0.018**	0	NA	1	1
*DBH*	cg25020204		rs117956167 (G/C)	136,466,632	–33,602	0.02	0	1	1	0.04	0.52	**0.043**	0.066
*PTEN*	cg01228636	TSS1500	rs140133143 (AT/A)	89,751,172	129,399	0	NA	1	1	0.04	0.53	**0.025**	**0.044**
			rs118039301 (A/C)	89,759,243	137,470	0	NA	1	1	0.04	0.53	**0.025**	**0.05**
	cg16687447	5′-UTR	rs185979581 (T/C)	88,920,089	–703,247	0.04	0.83	**0.007**	**0.014**	0	NA	1	1
*CHRM1*	cg13530039	TSS1500	rs2736595 (G/A)	61,720,538	–969,019	0.23	0.53	**0.035**	**0.034**	0.79	0.05	1	1
			rs2524294 (G/A)	61,721,234	–968,323	0.23	0.53	**0.035**	**0.045**	0.79	0.05	1	1
*NRXN3*	cg15572745	5′-UTR	rs144623790 (AAT/A)	78,026,973	–843,259	0.25	0.01	1	1	0.05	0.46	**0.044**	0.066
*CYP2A7*	cg25427638		rs10500282 (C/T)	41,508,442	119,085	0.39	0.56	**0.002**	**0.004**	0.29	0.27	0.65	0.787
			rs11673270 (C/A)	41,520,844	131,487	0.48	0.49	**0.009**	**0.018**	0.26	0.32	0.38	0.505
			rs3745275 (A/G)	41,531,705	142,348	0.42	0.59	**0.0001**	**0.002**	0.31	0.48	**0.014**	**0.028**
			rs3745277 (A/G)	41,531,915	142,558	0.48	0.54	**0.003**	**0.006**	0.22	0.43	0.058	0.088
			rs10409701 (A/G)	41,537,868	148,511	0.40	0.73	**0.0001**	**0.002**	0.21	0.52	**0.009**	**0.018**

Genomic positions are based on the NCBI Build 37/hg19 assembly. Significant region- and pheno-wide p-values are given in bold. SNP annotations were obtained from HaploReg v4.1 (http://www.broadinstitute.org/mammals/haploreg/haploreg.php).

SNP, single nucleotide polymorphism; AA, African American; EA, European American; A1 Freq, allele frequency of A1; R^2^, regression r-squared; Region-wide p, corrected empirical p-value based on 10^3^ max (T) permutations with correction for the number of variants tested for cis associations at this CpG site; Pheno-wide p, region-wide p-value after correcting for the 107 CpG sites tested using augmentation multiple testing procedure.

Annotations from HaploReg v4.1 corroborated the regulatory potentials of the significant *cis*-mQTLs for *NRXN1* and *CYP2A7* (Table 2 and Supplementary Tables 4A,B). According to the core 15-state model from the Roadmap Epigenomics Project (12), it was predicted that 13 of the 41 variants in *NRXN1* would show significant association with either enhancer or both enhancer and promoter histone marks in human brain tissues. A core set of five chromatin marks (H3K4me3, H3K4me1, H3K36me3, H3K27me3, and H3K9me3) assayed in 127 epigenomes was concatenated to train a ChromHMM model and compute posterior probabilities of 15 chromatin states for each variant in the Roadmap Epigenomics Project (12). Additionally, almost all of the 41 variants except rs13031157, rs17573587, rs17514717, and rs13023341 were predicted to alter one or more of the regulatory motif indicatives of TF binding sites, among which rs17514766 was detected to bind to SP1 in H1-hESC cells (13). Moreover, 16 of the 41 variants showed one to three independent QTL hits in the genome-wide repository of associations between SNPs and phenotypes (GRASP)^4^ (25) either as mQTLs for the same CpG site (i.e., cg10917619) in the temporal cortex, frontal cortex, or caudal pons (22) or as loci significantly associated with blood metabolite concentrations or ratios (26). Four variants, i.e., rs6545187, rs7574611, rs13031157, and rs7594170, significantly affected *NRXN1* gene expression in nerve tibial tissues based on the GTEx Project results (14). Among them, rs7574611 was discovered as both a *cis*-mQTL and an eQTL, and rs7594170 was significantly associated with both the serum concentration of 17-dimethylurate and *NRXN1* gene expression levels in independent studies (14, 22, 26). According to NCBI dbSNP functional annotation, all of the 41 variants within the range of 121 Kb (distance between the two mQTLs furthest away) are intronic except for rs67661616, which is within the 5′- untranslated region (UTR) region of *NRXN1* (Supplementary Figure 2).

Unlike the *cis*-mQTLs for *NRXN1*, 12 (18%) of the 67 variants (among them, rs373754258 is missing for HaploReg annotation) for *CYP2A7* showed significant associations with promoter or enhancer histone marks or DNase-hypersensitive sites in human induced pluripotent stem cells (iPSCs), embryonic stem cells (ESCs), or H1-derived neuronal progenitor cultured cells (ESDRs) (Table 2 and Supplementary Tables 4A,B) (12). However, similar to *NRXN1*, 61 of the variants were predicted to change one or more of the regulatory motifs. Eight variants affected TF binding in ChIP-Seq experiments of the ENCODE Project (13). The involved TFs include glucocorticoid receptor (GR), the CCCTC-binding factor (CTCF), the cohesion ring complex subunit RAD21, transcriptional co-activating protein P300, signal transducer and activator of transcription 3 (STAT3), estrogen receptor alpha (ERalpha_a), forkhead box protein A1 (FOXA1), and *trans*-acting T-cell-specific TF GATA-Binding Protein 3 (GATA3). Regarding the GRASP results (25), associations between 15 variants and methylation of cg25427638 were replicated in cerebellum, caudal pons, and frontal and temporal cortex tissues of an independent study with 150 neurologically normal Caucasian subjects (22). The 67 variants for *CYP2A7* overlap with *CYP2B6* and *CYP2A7P1* in a region spanning 35 Kb (Supplementary Figures 3A,B). Looking at the associated variants’ effect sizes as measured by *R*^2^, the effects of these *cis*-mQTLs are larger in AAs than in EAs (Table 2).

### *Cis*-expression quantitative trait loci mapping

As shown in Table 3, six *cis*-eQTLs were detected in the AA sample for six genes based on region-wide significance. Two of these variants passed the phenotype-wide significance threshold: rs73386029 for *CACNA2D1* and rs139998364 for *HTR5A*. Fifty-six region-wide significant *cis*-eQTLs were identified in the EA sample for *BDNF* and *EGLN2*, among which 54 variants were significantly associated with the expression of *EGLN2*, measured by probe hHC023008 (Table 3). All but 1 of the 54 variants for *EGLN2*, forming one LD block, showed phenotype-wide significance as well (Supplementary Figure 1).

**TABLE 3:** Significant expression probe-SNP pairs with region- or phenotype (pheno)-wide *cis* associations in the AA and EA samples.

Ethnicity	Gene	Expression Probe Oligo ID	SNP ID (A1/A2)	Variant Position	Distance to TSS	A1 Freq	*R* ^2^	Region-wide *P*	Pheno-wide *P*
**AA (*N* = 94)**	*DRD1*	hHC010798	rs147731662 (T/C)	175600872	729,709	0.01	0.21	**0.046**	0.092
	*CACNA2D1*	hHC009943	rs73386029 (C/A)	82036439	–36,592	0.02	0.25	**0.017**	**0.034**
	*CHRM2*	hHC005116	rs324650 (A/T)	136693661	140,262	0.67	0.17	**0.042**	0.084
	*HTR5A*	hHC008651	rs139998364 (A/G)	155628744	766,134	0.01	0.46	**0.018**	**0.036**
	*DNM1*	hHC021993	rs3824415 (A/G)	130145624	–820,039	0.22	0.19	**0.034**	0.068
	*C14orf28*	hHR004782	rs78410784 (G/C)	45639985	273,478	0.08	0.18	**0.036**	0.072
**EA (*N* = 84)**	*BDNF*	hHC017923	rs72887755 (A/G)	27801789	58,493	0.02	0.22	**0.035**	0.062
			rs116860953 (G/A)	27930226	186,930	0.02	0.21	**0.031**	**0.037**
	*EGLN2*	hHC023008	rs4802088 (T/C)	41255768	–49,377	0.03	0.30	**0.002**	**0.004**
			rs34406232 (A/C)	41305530	385	0.02	0.29	**0.003**	**0.006**

Genomic positions are based on the NCBI Build 37/hg19 assembly. Significant region- and pheno-wide p-values are in bold. Variant annotations were obtained from HaploReg v4.1 (http://www.broadinstitute.org/mammals/haploreg/haploreg.php).

SNP, single nucleotide polymorphism; AA, African American; EA, European American; TSS, transcriptional start site; A1 Freq, allele frequency of A1; R^2^, regression r-squared; Region-wide p, corrected empirical p-value based on 10^3^ max (T) permutations with correction for the number of variants tested for cis associations with this expression probe; Pheno-wide p, region-wide p-value after correcting for the 51 expression probes tested using augmentation multiple testing procedure.

Among the 50 *cis*-eQTLs for *EGLN2*, 23 showed chromatin states of promoter, enhancer, or DNase I activity cluster in human brain tissues (Table 3 and Supplementary Table 4C). One or more regulatory motifs were changed for 48 of the 54 variants (27). DNA polymerase II (POL2) and c-Myc binding sites were influenced by 15 variants in different cell lines based on the ENCODE Project (13).

### Connection of genetic variation, methylation, expression, and smoking status

**FIGURE 1:**
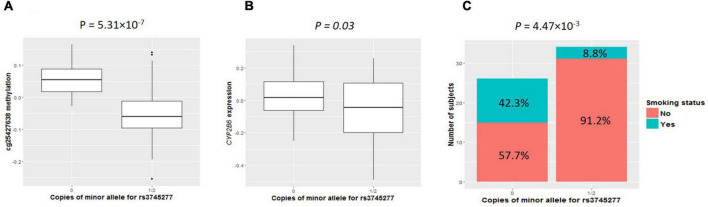
Comparisons of cg25427638 methylation, *CYP2B6* expression, and smoker percentage between subjects with zero copies of rs3745277 minor allele and those with either one or two copies. The two boxplots indicate methylation at cg25427638 **(A)** and expression for *CYP2B6* **(B)** for subjects with 0 or 1/2 copies of rs3745277 minor allele. **(A)** Student’s *t*-test results are included. Because of the existence of outliers, as shown in the top panel, robust statistical methods were implemented to confirm the *t*-test results. The barplot in **(C)** illustrates smoker and non-smoker percentages for each genotype. Fisher’s exact test was performed to obtain the *p*-value for this panel. All the *p*-values are significant (*P* < 0.05).

**FIGURE 2:**
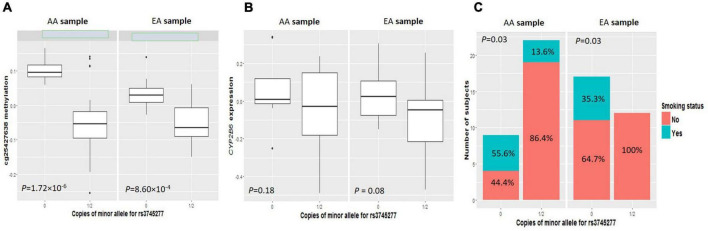
Comparisons of cg25427638 methylation, *CYP2B6* expression, and smoker percentage between subjects with zero copies of rs3745277 minor allele and those with either one or two copies in AAs or EAs, respectively. The four boxplots indicate methylation at cg25427638 **(A)** and expression for *CYP2B6* **(B)** for subjects with 0 or 1/2 copies of rs3745277 minor allele **(A)** in the AA (left) or EA (right) ethnic group. Student’s *t*-test results are included. The barplots in **(C)** illustrate smoker and non-smoker percentages for each genotype group in AAs or EAs. Fisher’s exact test was performed to obtain the *p*-values for this panel. *P*-values < 0.05 are considered significant.

As shown in clustering of *cis*-mQTLs in *CYP2B6* and *CYP2A7P1*, significant correlation between the mQTL-associated methylation site at *CYP2A7* (cg25427638) and *CYP2B6* expression and significantly different cg25427638 methylation and *CYP2B6* expression between smokers and non-smokers were observed. We picked one genotyped variant (rs3745277), which had strong biological evidence based on HaploReg v4.1 (28) results (Table 3), to check simultaneously the effect of its minor allele on cg25427638 methylation, *CYP2B6* expression, and smoking status. As shown in Figure 1, subjects with one or two copies of the minor allele (A) of rs3745277 (dominant effect) had a significant decrease in their methylation at cg25427638 (*P* = 5.31 × 10^–7^), a weak decrease in their expression of *CYP2B6* (*P* = 0.03), and a significantly lower percentage of smokers (8.8 vs. 42.3%; OR [95% CI] = 0.14 [0.02–0.62]; *P* = 4.47 × 10^–3^). We performed the same sets of analysis for AAs and EAs separately, and similar patterns of results were observed (Figure 2).

## Discussion

In this study, we performed *cis*-eQTL and mQTL analysis for 56 smoking susceptibility genes in human brain. Six and two *cis*-eQTLs were detected for *DRD1*, *CACNA2D1*, *CHRM2*, *HTR5A*, *DNM1*, and *C14orf28* in the AA sample and for *BDNF* and *EGLN2* in the EA sample, respectively. Except for *TAS2R38*, which has two detected *cis*-mQTLs, one *cis*-mQTL for each gene was found for *PDE1C*, *CHRM2*, *CHRNA2*, *PTEN*, *CHRM1*, and *CYP2A7* in the AA sample; and six *cis*-mQTLs were located for genes *NRXN1*, *TAS2R38*, *DBH*, *PTEN*, *NRXN3*, and *CYP2A7* in the EA sample, respectively. Only the *cis*-mQTL for *CYP2A7* is the same across the two ethnic samples. Among the QTLs determined, the *cis*-eQTL for *EGLN2* accounts for 54 (87%) of the 62 total significant variants for all the *cis*-eQTLs; the *cis*-mQTLs for *NRXN1* and *CYP2A7*, respectively, make up 42 (30%) and 68 (49%) of the 138 significant *cis*-mQTLs.

All 50 significantly associated variants within the *cis*-eQTL of *EGLN2* had strong biological evidence that it affected gene expression according to HaploReg v4.1 (Supplementary Table 4C). For example, rs34406232 is at an active transcriptional start site (TSS) not only in neuronal progenitors, neurons, and astrocytes (12, 13), but also in eight human brain regions including dorsolateral prefrontal cortex (12). It is also within a DNase I-hypersensitive site, which is characterized by open and accessible chromatin for active genes. More importantly, in H1-hESC cells, this variant interacts with POL2 (13) and changes a regulatory motif for Evi-1 (27), which positively regulates transcription from the POL2-binding promoter. Additionally, rs34406232 resides in the 5′-UTR region of *EGLN2* based on dbSNP annotation, and recent studies suggest that transcriptional regulation near the 5′-ends of genes exerts the strongest control of gene expression (7). All this evidence makes rs34406232 highly likely to be a functional regulatory variant for *EGLN2* expression. However, because nearly all the 54 *cis*-eQTLs are in complete LD (assessed by D’) with each other (Supplementary Figure 1), and they may cause changes in *EGLN2* mRNA expression individually or collectively, at present we cannot pin down rs34406232 as the causal variant.

When we compared *EGLN2* expression in smokers and non-smokers, no significant difference was detected, which is not surprising, because variants in *EGLN2* are reported to be associated with cigarettes smoked per day (CPD), breath carbon monoxide (CO), ND, smoking efficiency (CO/CPD), and nicotine metabolite ratio (NMR) (29–31) but not directly with smoking status. Bloom et al. (30) indicated that multiple SNPs in high LD with rs3733829, a genome-wide association study (GWAS) hit but without clear functional consequence prediction (29), are co-localized in the 5′-UTR or promoter region and may have impact on *EGLN2* gene transcription in subjects of European descent. It is even more interesting that Clark et al. (32) captured the *EGLN2* region followed by next-generation deep sequencing in 363 individuals of European ancestry and identified variant sets with regulatory annotations such as promoters and chromatin states involving or flanking active TSSs significantly associated with CPD. Eight of the 50 *cis*-eQTLs for *EGLN2* in this study were detected by Clark et al. with similar MAFs (32) and were included in both individual and sets of variants analysis. However, significant association with CPD was found only for different variant sets. These results provided us great confidence in the imputation quality of IMPUTE2 (33) for low-frequency variants and encourage us to believe that the *cis*-eQTLs determined here affect *EGLN2* gene transcription in aggregate, which then influences CPD or other smoking measures mentioned above.

Although the 41 *cis*-mQTLs for *NRXN1* were overrepresented with enhancer and promoter histone marks in human brain tissues (12), 36% of the variants were replicated as mQTLs for the same CpG site in three human brain regions by an independent study (22), and four of them affected *NRXN1* gene expression in tibial nerve tissues (14). None of these variants was identified as *cis*-eQTL in this study (Supplementary Table 4A). We did not detect a correlation between methylation at cg10917619 and *NRXN1* gene expression nor a difference of methylation at this site between smokers and non-smokers. The two non-synonymous rare variants [p.R206L and rs77665267 (p.T274P)], reported by our group as having an aggregate effect on smoking status in 1430 unrelated EA subjects, are 903 Kb away from the *cis*-mQTL, which are more likely to affect the encoded protein sequence directly (34). The SNP rs2193225 and a major haplotype that includes this SNP, 32 Kb downstream from the *cis*-mQTL, were significantly associated with different ND measures in an EA family sample with 671 subjects (35). Bierut et al. (36) nominated three SNPs (rs12623467, rs12467557, and rs10490162) within the mQTL region to be associated with ND status in smokers.

The *cis*-mQTL for *CYP2A7* was identified in both AA and EA samples. Fifteen of the variants were replicated for the same CpG site in four human brain regions by an independent study (22). But unlike the QTLs for *EGLN2* and *NRXN1*, this QTL is 116 Kb away from the CpG site (cg25427638), not overlapping with the target gene *CYP2A7*. Yet architectural proteins including cohesin (subunit RAD21) and CTCF were seen to be bound at several loci of the *cis*-mQTL, which can bring enhancers and promoters together that are located far apart in linear sequence (37). When we tried to correlate methylation at cg25427638 with gene expression, *CYP2B6* instead of *CYP2A7* and *CYP2A6* showed statistical significance (*r^2^* = 0.37; *P* = 0.004). The specific mechanisms of the biological effect of the *cis*-mQTL cg25427638 on DNA methylation in *CYP2A7* and *CYP2B6* gene expression are not clear. Further, *CYP2B6* expression was significantly different between smokers and non-smokers, whereas methylation of cg25427638 indicated a nominally significant difference. Observation of these differences may benefit from a larger sample, as no difference was found in the frontal cortex in a previous study of 26 subjects (38). By using one genotyped variant with strong biological evidence (rs3745277), we demonstrated its effect on cg25427638 methylation, *CYP2B6* expression, and smoking status simultaneously. Other variants within the *cis*-mQTL are expected to have similar regulatory effects on methylation, expression, and smoking status because of the high LD among the 68 variants. As in the *cis*-eQTL for *EGLN2*, we cannot identify causal variant(s) within the region.

Several variants in this region are reported to be associated with either CPD or nicotine metabolism, including rs4105144, a CPD GWAS hit (39); rs7260329, an NMR GWAS hit (31); and rs8109525, associated with nicotine metabolism independent of the well-studied non-synonymous variants rs3211371, rs3745274, and rs2279343 (CYP2B6*5 and *6) (40). Additionally, 16 CpG sites within 19q13 were affected by the NMR GWAS hit (rs7260329) using whole blood DNA (31), and rs8109525 was associated with differences in *CYP2B6* mRNA expression in liver biopsy samples (40). However, neither rs7260329 nor rs8109525 was in LD with the *cis*-mQTL in the BrainCloud cohort; rs4105144 was not available. None of the three variants was in LD with the mQTL in either the 1000 Genomes AFR or the EUR samples. Regulatory mechanism differences among tissues (blood, brain, and liver in this case) may explain the discrepancy (7). The three functional variants (rs3211371, rs3745274, and rs2279343) unfortunately were not available in this study either. Even though rs2279343 and rs3211371 were not in LD with the mQTL in the AFR and EUR samples of the 1000 Genomes Project, rs3745274 was found to be in high LD (*r*^2^ ≥ 0.8) with 4 and 8 mQTLs in the 1000 Genomes AFR and EUR samples, respectively. It is noted that two missense SNPs rs3745274 and rs2279343 were reported to be linked, and these two functional variants were observed to affect expression and activity of *CYP2B6* gene in liver (41), but the *cis*-mQTL could have an independent effect from them on *CYP3B6* gene expression in human brain.

There are several limitations of this study. First is sample size although even using modest sample sizes (60–100 individuals), early studies found a large number of genetic associations with differences in gene regulation (7). We acknowledge that more *cis*-eQTLs and mQTLs are anticipated if larger samples become available. Second is the inability to map *trans*-regulatory variants, although heritability studies suggest that more than half of the genetically explained variance in gene expression is attributable to *trans*-acting variants (42). However, we have to acknowledge that reliable detection of *trans*-QTLs has been challenging in humans because of the smaller effect of *trans*-acting variants compared with *cis*-QTLs and a higher statistical penalty for multiple testing (7). Besides enlarging the sample size, focusing on QTLs affecting expression of putative *trans*-regulatory elements (thereby minimizing the number of tests performed) might be another promising approach (7). Third, although post-mortem prefrontal cortex gray matter tissue homogenates were used to measure mRNA expression and DNA methylation in the BrainCloud cohort, a highly relevant brain region for studying smoking (43–45), previous research has shown that active regulatory regions and non-coding transcripts often are cell- and tissue-specific, and regulatory mechanism differences between tissues or different cell types are of a much larger magnitude (7). Considering that the physiological response to nicotine is a complex process involving multiple brain regions (46) and that the brain is built from a large number of cell types (47), in-depth investigation of gene regulation in different brain regions and cell types is anticipated, for example by using the PsychENCODE data (48). Fourth, we employed a relatively simple and straightforward group comparison to reveal those significant eQTLs and mQTLs, which might be not as robust as we wish as such approach could not well control the potential confounders if they might exist in the data. Finally, the smoking status phenotype derived from two variables in the original study is primitive. The limited sample size of this study may magnify the sampling error for this phenotype, and statistical power may be reduced to connect molecular measurements with smoking status compared with other more refined and commonly used ND measures, e.g., CPD, FTND, and ND classification based on the Diagnostic and Statistical Manual of Mental Disorders (DSM).

Despite these limitations, this is one of a few studies integrating data on genetic variation, DNA methylation, mRNA expression, and smoking phenotypes for the same cohort. The connection we found among *cis*-mQTL, CpG site methylation in *CYP2A7*, *CYP2B6* expression, and smoking status received vigorous support from projects enabling parallel sequencing of regulatory features; i.e., the Roadmap Epigenomics, ENCODE, and GTEx projects. Further studies are warranted to test causal relations among these regulatory layers or to weave other factors into the picture such as chromatin accessibility, histone modifications, and TF binding using one cohort if possible. In this way, we are able not only to learn about mechanistic steps between genetic variation and smoking effectively, but also to narrow down the GWAS variant list efficiently.

## Data availability statement

The dataset used for the analysis described in this manuscript was obtained from dbGaP at http://www.ncbi.nlm.nih.gov/gap through dbGaP accession number phs000417.v2.p1. Submission of the data, phs000417.v2.p1, to dbGaP was provided by Barbara Lipska and Joel Kleinman. Collection of the data was through a collaborative study sponsored by the NIMH Intramural Research Program. Initial report on this dataset was from Colantuoni et al. (15).

## Author contributions

ZY, JY, YM, and ML participated in data analysis. ZY, JY, and ML participated in manuscript writing and editing. ML conceived the study and involved in every step of this study. All authors approved the manuscript as submitted.

## Footnotes

1
http://www.libd.org/braincloud

2
https://mathgen.stats.ox.ac.uk/impute/1000GP_Phase3.html

3
http://www.internationalgenome.org/1000-genomes-browers/index.html

4
http://grasp.nhlbi.nih.gov/Overview.aspx

## Supplementary material

Supplementary Figure 1 Linkage disequilibrium (LD) D’ plot of the 54 eQTLs for *EGLN2* gene in BrainCloud EA sample. The LD D’ plot was drawn using Haploview (https://www.broadinstitute.org/scientific-community/science/programs/medical-and-population-genetics/haploview/haploview). Gene annotation and regulatory feature tracks were obtained from the 1000 Genomes Browser (http://www.internationalgenome.org/1000-genomes-browers/index.html). The black vertical bar indicates position of expression probe hHC023008. Red boxes highlight eight rare variants, which were found to be collectively affecting smoking quantity by Clark et al. (32). The yellow star marks the variant with the strongest biological evidence based on HaploReg v4.1 results (http://www.broadinstitute.org/mammals/haploreg/haploreg.php).

Supplementary Figure 2 Linkage disequilibrium (LD) D’ plot of the 42 mQTLs for *NRXN1* gene in BrainCloud EA sample. The LD D’ plot was drawn using Haploview (https://www.broadinstitute.org/scientific-community/science/programs/medical-and-population-genetics/haploview/haploview). Gene annotation and regulatory features tracks were obtained from the 1000 Genomes Browser (http://www.internationalgenome.org/1000-genomes-browers/index.html). Black vertical bar indicates position of methylation probe cg10917619.

Supplementary Figure 3 Linkage disequilibrium (LD) D’ plot of the 68 mQTLs for *CYP2A7* in BrainCloud **(A)** African American (AA) sample, and **(B)** European American (EA) sample. The LD D’ plots were drawn using Haploview (https://www.broadinstitute.org/scientific-community/science/programs/medical-and-population-genetics/haploview/haploview). Gene annotation and regulatory features tracks were obtained from the 1000 Genomes Browser (http://www.internationalgenome.org/1000-genomes-browers/index.html). Black vertical bars indicate position of methylation probe cg25427638. Yellow star marks variant with strongest biological evidence based on HaploReg v4.1 results (http://www.broadinstitute.org/mammals/haploreg/haploreg.php).
