# Supplementary material for: Investigation of the genetic effect of 56 tobacco-smoking susceptibility genes on DNA methylation and RNA expression in human brain
Source: Front Psychiatry. 2022 Aug 18;13:924062. doi: 10.3389/fpsyt.2022.924062 (PMC9433921; doi:10.3389/fpsyt.2022.924062)
Supplement: Supplementary file 1 [file Data_Sheet_1.docx]

**Supplementary Table 1.** Information on 56 genes located in 46 genetic susceptibility loci for nicotine dependence

| Locus No. | Gene(s) | Chr | Linkage (distance)/GWAS studies | Reference(s) |
| --- | --- | --- | --- | --- |
| 1 | *CHRNB2* | 1q21.3 |  | [1] |
| 2 | *NRXN1* | 2p16.3 | GWAS | [2; 3; 4; 5] |
| 3 | *GABRA2*/*A4* | 4p12 | Within the nominated linkage peak on 4p15-q13.1 (0 bp) | [6; 7; 8] |
| 4 | *CHRNA9* | 4p14 |  | [4] |
| 5 | *DRD1* | 5q35.2 | Within the nominated linkage peak on 5q34-q35 (0 bp) | [9] |
| 6 | *OPRM1* | 6q25.2 | Within the nominated linkage peak on 6q23.3-q27 (0 bp) | [6; 10] |
| 7 | *MAP3K4* | 6q26 | Within the nominated linkage peak on 6q23.3-q27 (0 bp) | [11] |
| 8 | *DDC* | 7p12.1 |  | [12; 13] |
| 9 | *HTR5A* | 7q36.2 | Close to the nominated linkage peak on 7q31.2-q36.1 (4.2 Mbp) | [6] |
| 10 | *CHRM2* | 7q33 | Within the nominated linkage peak on 7q31.2-q36.1 (0 bp) | [11] |
| 11 | *CACNA2D1* | 7q21.11 | GWAS | [14] |
| 12 | *PDE1C* | 7p14.3 | GWAS | [15] |
| 13 | *TAS2R38* | 7q34 | Within the nominated linkage peak on 7q31.2-q36.1 (0 bp) | [4; 16; 17] |
| 14 | *CHRNA2* | 8p21.2 |  | [4] |
| 15 | *CHRNB3*/*A6* | 8p11.21 | GWAS | [5; 15; 18; 19; 20; 21; 22; 23; 24; 25; 26] |
| 16 | *CSGALNACT1*-*INTS10* | 8p21.3 | GWAS | [14] |
| 17 | *DLC1* | 8p22 | GWAS | [14] |
| 18 | *DBH* | 9q34.2 | GWAS | [4; 6; 11; 27; 28; 29] |
| 19 | *GABBR2* | 9q22.33 | Within the nominated linkage peak on 9q21.33-q33 (0 bp) | [4; 30] |
| 20 | *GRIN3A* | 9q31.1 | Within the nominated linkage peak on 9q21.33-q33 (0 bp) | [4; 31] |
| 21 | *NTRK2* | 9q21.33 | GWAS,[32] close to the nominated linkage peak on 9q21.33-q33 (2.7 Mbp) | [4; 33] |
| 22 | *SHC3* | 9q22.1 | Within the nominated linkage peak on 9q21.33-33 (0 bp) | [34] |
| 23 | *DNM1* | 9q34.11 | Close to the nominated linkage peak on 9q21.33-33 (3.1 Mbp) | [4; 35] |
| 24 | *CHAT* | 10q11.23 |  | [36] |
| 25 | *PTEN* | 10q23.1 | Within the nominated linkage peak on 10q21.2-q26.2 (0 bp) | [37] |
| 26 | *NRG3* | 10q23.1 | Within the nominated linkage peak on 10q21.2-q26.2 (0 bp) | [38] |
| 27 | *LOC100188947* | 10q23.32 | GWAS | [27] |
| 28 | *DRD4* | 11p15.5 | Within the nominated linkage peak on 11p15-q13.4 (1.3 Mbp) | [39; 40; 41] |
| 29 | *TTC12-ANKK1-DRD2* | 11q23.2 | Within the modest linkage peak on 11q23 (0 bp) | [4; 6; 42; 43; 44; 45; 46; 47; 48; 49; 50; 51] |
| 30 | *HTR3A* | 11q23.2 | Within the modest linkage peak on 11q23 (0 bp) | [51; 52] |
| 31 | *NRXN2* | 11q13.1 |  | [4] |
| 32 | *CHRM1* | 11q12.3 | Within the nominated linkage peak on 11p15-q13.4 (0 bp) | [53] |
| 33 | *BDNF* | 11p14.1 | GWAS,[27] within the nominated linkage peak on 11p15-q13.4 (0 bp) | [54; 55] |
| 34 | *ARRB1* | 11q13.4 | Within the nominated linkage peak on 11p15-q13.4 (1.5 Mbp) | [56] |
| 35 | *APBB1* | 11p15.4 | Within the nominated linkage peak on 11p15-q13.4 (0 bp) | [57] |
| 36 | *GRIN2B* | 12p13.1 | GWAS,[32] close to the modest linkage peak on 12p13.31-p13.32 (3.6 Mbp) | [11; 58] |
| 37 | *NRXN3* | 14q24.3 |  | [4] |
| 38 | *C14orf28* | 14q21.2 | GWAS | [14] |
| 39 | *PPP1R1B* | 17q12 | Within the nominated linkage peak on 17p13.1-q22 (0 bp) | [59] |
| 40 | *DLG4-GABARAP* | 17p13.1 | Within the nominated linkage peak on 17p13.1-q22 (0 bp) | [60] |
| 41 | *SLC6A4* | 17q11.2 | Within the nominated linkage peak on 17p13.1-q22 (0 bp) | [48; 61; 62] |
| 42 | *CHRNB1* | 17p13.1 | Close to the nominated linkage peak on 17p13.1-q22 (3.2 Mbp) | [11; 53] |
| 43 | *ARRB2* | 17p13.2 |  | [4; 56] |
| 44 | *RAB4B*-*EGLN2-CYP2A6*/*A7*/*B6* | 19q13.2 | GWAS | [6; 23; 48; 63; 64; 65] [15; 27; 66; 67] |
| 45 | *CHRNA4* | 20q13.33 | Close to the nominated linkage peak on 20q13.12-q13.32 (3.6 Mbp) | [1; 11; 68; 69; 70; 71; 72] |
| 46 | *COMT* | 22q11.21 | Close to the nominated linkage peak on 22q11.23-q12.1 (4.5 Mbp) | [4; 73; 74; 75; 76; 77; 78] |

**Notes:** Details about the gene selection procedure can be found in the Review of Yang and Li.[79] The “Linkage/GWAS studies” column indicates whether a gene is within (< 2Mbp) or close to (2-5 Mbp) any reported linkage region or detected in GWASs. All the linkage peaks are based on the consensus in the Review unless otherwise noted.

**Abbreviations:** Chr = chromosome; bp = base pair; Mbp = megabase pair; CAS = candidate gene association study; NGS = next-generation sequencing study.

**Supplementary Table 2.** Information on the 50 expression and 98 methylation probes for 56 genes located in 46 genetic susceptibility loci

| **Chr** | **Gene** | **Gene strand** | **Gene position** | **TSS coordinate** | **Expression** | | | **Methylation** | | | |
| --- | --- | --- | --- | --- | --- | --- | --- | --- | --- | --- | --- |
|  |  |  |  |  | **Illumina ID** | **Oligo ID** | **Probe position** | **Probe ID** | **CpG position** | **SNP in CpG?** | **CpG island location** |
| 1 | *CHRNB2* | + | 154540256-154552353 | 154540257 | HEEBO-065-HCC65I17 | hHC024785 | 154548409-154548478 | cg21052164 | 154539977 | N | 154539927-154540715 |
|  |  |  |  |  |  |  |  | cg00818872 | 154540271 | N |  |
| 2 | *NRXN1* | - | 50145643-51259674 | 51255603 | HEEBO-029-HCC29N11 | hHC011075 | 50147539-50147608 | cg16279786 | 51255306 | N | 51254462-51255631 |
|  |  |  |  |  |  |  |  | cg10917619 | 51255627 | N |  |
| 4 | *CHRNA9* | + | 40337468-40356973 | 40337469 | HEEBO-021-HCC21M22 | hHC007990 | 40356890-40356959 | cg10375110 | 40337443 | N |  |
|  |  |  |  |  |  |  |  | cg23621817 | 40337853 | N |  |
|  | *GABRA2* | - | 46251581-46391396 | 46391945 | HEEBO-029-HCC29D9 | hHC010833 | 46252316-46252385 | cg21820677 | 46392528 | N | 46391171-46392738 |
|  |  |  |  |  |  |  |  | cg15918284 | 46392657 | N |  |
|  | *GABRA4* | - | 46920916-46995580 | 46995580 | HEEBO-008-HCC8H13 | hHC002869 | 46921585-46921654 | cg03593419 | 46995474 | N | 46994956-46995967 |
|  |  |  |  |  |  |  |  | cg16358826 | 46996264 | N | 46996078-46996300 |
| 5 | *DRD1* | - | 174867674-174871163 | 174871163 | HEEBO-029-HCC29B22 | hHC010798 | 174868285-174868354 | cg16112129 | 174870968 | N | 174870500-174872470 |
|  |  |  |  |  |  |  |  | cg17307280 | 174871636 | N |  |
| 6 | *OPRM1* | + | 154360442-154440594 | 154360443 | HEEBO-045-HCC45I7 | hHC017095 | 154412509-154412578 | cg14262937 | 154360351 | N |  |
|  |  |  |  |  |  |  |  | cg22719623 | 154360732 | N | 154360422-154361116 |
|  | *MAP3K4* | + | 161412821-161538417 | 161412822 | HEEBO-106-HCA106B14 | hHA040358 | 161519390-161519459 | cg22685251 | 161413652 | Y | 161412230-161413698 |
| 7 | *PDE1C* | - | 31792692-32338941 | 32110991 | HEEBO-037-HCC37J17 | hHC014057 | 31792718-31792787 | cg22131691 | 32110988 | N | 32109753-32111164 |
|  |  |  |  |  |  |  |  | cg00546491 | 32111062 | N |  |
|  | *DDC* | - | 50526133-50633154 | 50628751 | HEEBO-015-HCC15F9 | hHC005505 | 50526277-50526346 | cg04144768 | 50628154 | N |  |
|  |  |  |  |  |  |  |  | cg03843951 | 50629634 | N |  |
|  | *CACNA2D1* | - | 81579417-82073031 | 82073031 | HEEBO-026-HCC26O7 | hHC009943 | 81579471-81579540 | cg04008901 | 82072779 | N | 82071945-82073635 |
|  |  |  |  |  |  |  |  | cg06379754 | 82073573 | N |  |
|  | *CHRM2* | + | 136553415-136704999 | 136553399 | HEEBO-014-HCC14F4 | hHC005116 | 136704457-136704526 | cg04748704 | 136553243 | N |  |
|  |  |  |  |  |  |  |  | cg00973677 | 136553595 | N | 136553295-136556346 |
|  | *TAS2R38* | - | 141672431-141673573 | 141673489 | NA | | | cg25481253 | 141673384 | Y |  |
|  |  |  |  |  |  |  |  | cg03017475 | 141674341 | N |  |
|  | *HTR5A* | + | 154862545-154877459 | 154862610 | HEEBO-023-HCC23I11 | hHC008651 | 154876848-154876917 | cg15835825 | 154862030 | N |  |
|  |  |  |  |  |  |  |  | cg25780543 | 154862770 | N |  |
| 8 | *DLC1* | - | 13072081-13372395 | 13372395 | HEEBO-092-HCA92G22 | hHA035110 | 13072198-13072267 | cg05226008 | 13372132 | N |  |
|  |  |  |  |  |  |  |  | cg00933411 | 13373090 | N |  |
|  | *CSGALNACT1* | - | 19261671-19460056 | 19460056 | HEEBO-006-HCC6J20 | hHC002156 | 19262195-19262264 | NA | | | |
|  | *INTS10* | + | 19674917-19709586 | 19674918 | HEEBO-025-HCC25M12 | hHC009516 | 19709413-19709482 | NA | | | |
|  | *CHRNA2* | - | 27317278-27336813 | 27336758 | NA | | | cg02953306 | 27336626 | Y |  |
|  |  |  |  |  |  |  |  | cg04953015 | 27338236 | Y |  |
|  | *CHRNB3* | + | 42552561-42592209 | 42552562 | HEEBO-027-HCC27C4 | hHC010036 | 42591751-42591820 | cg00367281 | 42552470 | Y |  |
|  |  |  |  |  |  |  |  | cg06840801 | 42552719 | N |  |
|  | *CHRNA6* | - | 42607779-42623619 | 42623619 | HEEBO-047-HCC47C4 | hHC017716 | 42608261-42608330 | cg07906724 | 42623946 | N |  |
| 9 | *NTRK2* | + | 87283465-87638505 | 87283466 | HEEBO-013-HCC13A17 | hHC004625 | 87636860-87636929 | cg22402007 | 87282823 | N | 87282549-87285901 |
|  |  |  |  |  |  |  |  | cg09539438 | 87283789 | N |  |
|  | *SHC3* | - | 91620686-91793682 | 91793682 | NA | | | cg13351583 | 91793648 | N | 91792490-91793763 |
|  |  |  |  |  |  |  |  | cg00420568 | 91794904 | N | 91794806-91795518 |
|  | *GABBR2* | - | 101050365-101471175 | 101471479 | HEEBO-023-HCC23B14 | hHC008486 | 101050909-101050978 | cg02058918 | 101470884 | N | 101470524-101472117 |
|  |  |  |  |  |  |  |  | cg07903918 | 101471986 | N |  |
|  | *GRIN3A* | - | 104331634-104500862 | 104500862 | HEEBO-017-HCC17D10 | hHC006226 | 104331813-104331882 | cg08997253 | 104500729 | N | 104499137-104501229 |
|  |  |  |  |  |  |  |  | cg18794577 | 104501030 | N |  |
|  | *DNM1* | + | 130965662-131017527 | 130965663 | HEEBO-058-HCC58E9 | hHC021993 | 131008704-131008773 | cg02494117 | 130965474 | N | 130965354-130966691 |
|  |  |  |  |  |  |  |  | cg13309018 | 130965761 | N |  |
|  | *DBH* | + | 136501484-136524466 | 136501485 | HEEBO-064-HCC64K10 | hHC024442 | 136523852-136523921 | cg25020204 | 136500234 | N |  |
|  |  |  |  |  |  |  |  | cg07824742 | 136501784 | Y | 136501723-136501928 |
| 10 | *CHAT* | + | 50822082-50873150 | 50817141 | HEEBO-099-HCA99O4 | hHA037972 | 50822104-50822173 | cg12052765 | 50816963 | N | 50816963-50820806 |
|  |  |  |  |  |  |  |  | cg18592174 | 50817306 | N |  |
|  | *NRG3* | + | 83635069-84746935 | 83635070 | HEEBO-025-HCC25D4 | hHC009292 | 84745521-84745590 | NA | | | |
|  | *PTEN* | + | 89623194-89728532 | 89623195 | HEEBO-028-HCC28K6 | hHR010614 | 89726288-89726357 | cg21480743 | 89621419 | N | 89621218-89624183 |
|  |  |  |  |  |  |  |  | cg01228636 | 89621773 | N |  |
|  |  |  |  |  |  |  |  | cg04738091 | 89622084 | N |  |
|  |  |  |  |  |  |  |  | cg21573601 | 89622589 | N |  |
|  |  |  |  |  |  |  |  | cg20849549 | 89623138 | N |  |
|  |  |  |  |  |  |  |  | cg16687447 | 89623336 | N |  |
|  |  |  |  |  |  |  |  | cg17489897 | 89623432 | N |  |
|  |  |  |  |  |  |  |  | cg08859916 | 89624102 | N |  |
|  | *LOC100188947* | - | 93066719-93371217 | 93371217 | NA | | | NA | | | |
| 11 | *DRD4* | + | 637304-640703 | 637305 | HEEBO-064-HCC64C11 | hHC024251 | 640629-640698 | cg06825142 | 637170 | N | 636306-637963 |
|  | *APBB1* | - | 6416354-6440300 | 6440644 | HEEBO-060-HCC60F2 | hHC022778 | 6416538-6416607 | cg19327844 | 6440482 | N | 6439781-6440887 |
|  |  |  |  |  |  |  |  | cg05079045 | 6440803 | N |  |
|  | *BDNF* | - | 27676441-27681196 | 27743296 | HEEBO-047-HCC47K19 | hHC017923 | 27676838-27676907 | cg27351358 | 27743258 | N | 27743206-27744913 |
|  |  |  |  |  |  |  |  | cg16257091 | 27743580 | N |  |
|  | *CHRM1* | - | 62676150-62689012 | 62689012 | HEEBO-051-HCC51K5 | hHC019445 | 62676441-62676510 | cg00987015 | 62688751 | N |  |
|  |  |  |  |  |  |  |  | cg13530039 | 62689557 | N |  |
|  | *NRXN2* | - | 64373645-64490660 | 64490660 | HEEBO-042-HCC42K22 | hHC016006 | 64373670-64373739 | cg16718678 | 64490633 | N | 64490169-64491216 |
|  | *ARRB1* | - | 74976481-75062873 | 75062875 | HEEBO-053-HCC53J15 | hHC020199 | 74979933-74980002 | NA | | | |
|  | *TTC12* | + | 113185328-113244016 | 113185329 | HEEBO-051-HCC51O24 | hHC019560 | 113243931-113244000 | cg12177743 | 113185079 | N | 113184925-113186066 |
|  |  |  |  |  |  |  |  | cg24264506 | 113185537 | N |  |
|  | *ANKK1* | + | 113258512-113271140 | 113258513 | HEEBO-056-HCC56P9 | hHC021489 | 113270929-113270998 | NA | | | |
|  | *DRD2* | - | 113280316-113346001 | 113345881 | HEEBO-051-HCC51C1 | hHC019249 | 113280823-113280892 | cg12758687 | 113346327 | N | 113344914-113346439 |
|  |  |  |  |  |  |  |  | cg21330703 | 113346388 | N |  |
|  | *HTR3A* | + | 113845909-113861034 | 113845910 | HEEBO-106-HCA106I12 | hHA040524 | 113857476-113857545 | cg24134767 | 113845638 | N |  |
| 12 | *GRIN2B* | - | 13714409-14133022 | 14133052 | HEEBO-060-HCC60E20 | hHC022772 | 13714915-13714984 | cg04016326 | 14132940 | N |  |
|  |  |  |  |  |  |  |  | cg13264741 | 14133426 | N | 14133083-14135400 |
| 14 | *C14orf28* | + | 45366506-45376460 | 45366507 | HEEBO-013-HCC13H6 | hHR004782 | 45376220-45376289 | NA | | | |
|  | *NRXN3* | + | 78870092-80330760 | 78870093 | HEEBO-027-HCC27P15 | hHC010359 | 80328681-80328750 | cg16372520 | 78869751 | N |  |
|  |  |  |  |  |  |  |  | cg15572745 | 78870232 | N |  |
| 17 | *ARRB2* | + | 4613788-4624795 | 4613789 | HEEBO-038-HCC38E24 | hHC014328 | 4624637-4624706 | cg03950654 | 4613328 | N | 4612478-4614747 |
|  |  |  |  |  |  |  |  | cg23779331 | 4614312 | N |  |
|  | *DLG4* | - | 7093211-7123369 | 7123030 | HEEBO-069-HCC69C6 | hHC026166 | 7093896-7093965 | cg12228229 | 7122261 | N |  |
|  |  |  |  |  |  |  |  | cg02740128 | 7123860 | N | 7122940-7123898 |
|  | *GABARAP* | - | 7143737-7145753 | 7145753 | HEEBO-071-HCC71M23 | hHR027191 | 7145576-7145645 | cg25737491 | 7145532 | N | 7145374-7146069 |
|  |  |  |  |  |  |  |  | cg23983449 | 7146757 | N | 7146209-7146827 |
|  | *CHRNB1* | + | 7348406-7360932 | 7348406 | NA | | | cg04809787 | 7348339 | N | 7348187-7349319 |
|  |  |  |  |  |  |  |  | cg18884137 | 7348490 | Y |  |
|  | *SLC6A4* | - | 28521337-28562986 | 28562705 | NA | | | cg22584138 | 28562220 | N | 28562022-28563220 |
|  |  |  |  |  |  |  |  | cg05016953 | 28562813 | N |  |
|  | *PPP1R1B* | + | 37783178-37792877 | 37783179 | HEEBO-106-HCA106C7 | hHA040375 | 37783656-37783725 | cg00112517 | 37783011 | N |  |
|  |  |  |  |  |  |  |  | cg08411435 | 37784024 | N | 37783122-37784064 |
| 19 | *RAB4B* | + | 41284170-41302847 | 41284177 | HEEBO-064-HCC64A23 | hHC024215 | 41292785-41292854 | cg24958765 | 41283667 | N | 41283544-41284469 |
|  |  |  |  |  |  |  |  | cg13332130 | 41284234 | N |  |
|  | *EGLN2* | + | 41305047-41314337 | 41305145 | HEEBO-060-HCC60O16 | hHC023008 | 41314080-41314149 | cg22499964 | 41304369 | N | 41304039-41304401 |
|  |  |  |  |  |  |  |  | cg22671726 | 41305423 | N | 41304543-41305934 |
|  | *CYP2A6* | - | 41349442-41356352 | 41356340 | HEEBO-071-HCC71I21 | hHR027093 | 41349588-41349657 | cg05910970 | 41355973 | N |  |
|  |  |  |  |  |  |  |  | cg02043477 | 41357152 | N |  |
|  | *CYP2A7* | - | 41381343-41388657 | 41388657 | HEEBO-066-HCC66P15 | hHR025335 | 41381465-41381534 | cg20075229 | 41388937 | N |  |
|  |  |  |  |  |  |  |  | cg25427638 | 41389357 | Y |  |
|  | *CYP2B6* | + | 41497203-41524301 | 41497204 | HEEBO-087-HCA87O20 | hHA033380 | 41518580-41518649 | cg10322876 | 41496749 | N |  |
|  |  |  |  |  |  |  |  | cg19756068 | 41497222 | N |  |
| 20 | *CHRNA4* | - | 61974664-61992695 | 61992748 | HEEBO-048-HCC48I6 | hHC018246 | 61976458-61976527 | cg00318573 | 61993118 | N | 61992084-61993608 |
|  |  |  |  |  |  |  |  | cg08912400 | 61993427 | N |  |
| 22 | *COMT* | + | 19929262-19957496 | 19929309 | HEEBO-086-HCA86H14 | hHA032822 | 19929315-19929384 | cg15926585 | 19928445 | Y |  |

**Notes:** Whenever available, information in this table are based on the BrainCloud and BrainCloudMethyl applications distributed through the BrainCloud project website (<http://braincloud.jhmi.edu/>), with genomic positions lifted from NCBI Build 36/hg18 to Build 37/hg19. Otherwise, gene positions and TSS coordinates were obtained from the UCSC Genome Browser (<https://genome.ucsc.edu/>) and the Database of Transcriptional Start Sites (<http://dbtss.hgc.jp/>), respectively. All the genomic positions in this table are based on the NCBI Build 37/hg19 assembly.

**Abbreviations:** Chr = chromosome; TSS = transcriptional start site; NA = not available.

**Supplementary Table 3.** Information on the imputation intervals for each of the 56 genes except for *LOC100188947* gene

| **Chr** | **Imputation interval** | **Gene(s) within the interval** | **Number of variations** | |
| --- | --- | --- | --- | --- |
|  |  |  | **Before imputation** | **After imputation (info≥0.3)** |
| 1 | 153539977-155552353 | *CHRNB2* | 358 | 15,235 |
| 2 | 49145643-52259674 | *NRXN1* | 1,162 | 36,333 |
| 4 | 39337443-41356973 | *CHRNA9* | 557 | 20,469 |
|  | 45251581-47996264 | *GABRA2*, *GABRA4* | 518 | 26,244 |
| 5 | 173867674-175871636 | *DRD1* | 694 | 17,969 |
| 6 | 153360351-155440594 | *OPRM1* | 746 | 22,384 |
|  | 160412821-162538417 | *MAP3K4* | 845 | 23,280 |
| 7 | 30792692-33338941 | *PDE1C* | 964 | 26,573 |
|  | 49526133-51633154 | *DDC* | 607 | 21,068 |
|  | 80579417-83073573 | *CACNA2D1* | 804 | 24,745 |
|  | 135553243-137704999 | *CHRM2* | 660 | 19,723 |
|  | 140672431-142674341 | *TAS2R38* | 491 | 17,695 |
|  | 153862030-155877459 | *HTR5A* | 819 | 20,892 |
| 8 | 12072081-14373090 | *DLC1* | 930 | 32,877 |
|  | 18261671-20709586 | *CSGALNACT1*, *INTS10* | 1,246 | 32,649 |
|  | 26317278-28338236 | *CHRNA2* | 851 | 21,668 |
|  | 41552470-43623946 | *CHRNB3*, *CHRNA6* | 301 | 19,679 |
| 9 | 86282823-88638505 | *NTRK2* | 683 | 21,899 |
|  | 90620686-92794904 | *SHC3* | 575 | 21,137 |
|  | 100050365-102471986 | *GABBR2* | 656 | 20,634 |
|  | 103331634-105501030 | *GRIN3A* | 750 | 24,036 |
|  | 129965474-132017527 | *DNM1* | 465 | 16,868 |
|  | 135500234-137524466 | *DBH* | 769 | 20,960 |
| 10 | 49816963-51873150 | *CHAT* | 585 | 15,838 |
|  | 82635069-85746935 | *NRG3* | 981 | 31,941 |
|  | 88621419-90728532 | *PTEN* | 599 | 17,687 |
| 11 | 0-1640703 | *DRD4* | 317 | 16,202 |
|  | 5416354-7440803 | *APBB1* | 1,023 | 22,709 |
|  | 26676441-28743580 | *BDNF* | 509 | 16,158 |
|  | 61676150-65490660 | *CHRM1*, *NRXN2* | 677 | 32,854 |
|  | 73976481-76062873 | *ARRB1* | 524 | 19,121 |
|  | 112258512-114861034 | *TTC12*, *ANKK1*, *DRD2*, *HTR3A* | 886 | 24,711 |
| 12 | 12714409-15133426 | *GRIN2B* | 889 | 23,136 |
| 14 | 44366506-46376460 | *C14orf28* | 441 | 19,237 |
|  | 77869751-81330760 | *NRXN3* | 1,141 | 33,808 |
| 17 | 3613328-5624795 | *ARRB2* | 660 | 20,915 |
|  | 6093211-8360932 | *DLG4*, *GABARAP*, *CHRNB1* | 685 | 21,292 |
|  | 27521337-29562986 | *SLC6A4* | 239 | 16,612 |
|  | 36783011-38792877 | *PPP1R1B* | 369 | 16,949 |
| 19 | 40283667-42524301 | *RAB4B*, *EGLN2*, *CYP2A6*, *CYP2A7*, *CYP2B6* | 429 | 21,151 |
| 20 | 60974664-62993427 | *CHRNA4* | 444 | 20,255 |
| 22 | 18928445-20957496 | *COMT* | 469 | 16,689 |

**Notes:** All the imputation intervals in this table are based on the NCBI Build 37/hg19 assembly.

**Abbreviations:** Chr = chromosome; info = the metric used by IMPUTE2 to measure imputation quality.

**Supplementary Table 4A.** HaploReg annotations for the significant cis-regulatory variants detected in *NRXN1* on chromosome 2

| Pos (hg38) | SNP ID | Promoter histone marks | Enhancer histone marks | DNA Source | Protein bound | Motifs changed | Selected eQTL hit | RefSeq gene | dbSNP func annot |
| --- | --- | --- | --- | --- | --- | --- | --- | --- | --- |
| 50956233 | rs56159305 | PANC | BRST, STRM, BRN |  |  | HNF1 |  | *NRXN1* | intronic |
| 50962097 | rs70958634 |  | ESDR, ESC, PANC |  |  | AP-3, Ik-2, Pou3f2 |  | *NRXN1* | intronic |
| 50965967 | rs17572910 |  |  |  |  | Evi-1, Foxp1, KAP1 |  | *NRXN1* | intronic |
| 50971246 | rs7567632 |  |  |  |  | Cart1, Foxj2, HNF4 |  | *NRXN1* | intronic |
| 50971500 | rs7567909 |  |  |  |  | HNF4, Nkx2, RXRA, SREBP, ZEB1 |  | *NRXN1* | intronic |
| 50971537 | rs1985832 |  |  |  |  | Gfi1, p300 |  | *NRXN1* | intronic |
| 50972094 | rs201612470 |  | ESC |  |  | Cart1, Dbx1, Foxa, Foxd3, Foxj2, Foxo, Foxp1, HDAC2, Hoxd8, Nanog, Nkx6-1, Pou6f1, Zfp105, p300 |  | *NRXN1* | intronic |
| 50972414 | rs13008752 |  | ESC |  |  | ERalpha-a, Esr2 |  | *NRXN1* | intronic |
| 50973408 | rs6545187 |  | ESC, IPSC, PANC |  |  | Hltf, SRF, YY1 | 1 hit | *NRXN1* | intronic |
| 50973999 | rs11308522 |  | IPSC, PANC |  |  | Foxa, Foxp1, Zfp105 |  | *NRXN1* | intronic |
| 50976422 | rs12621330 |  | ESDR, ESC |  |  | Maf, NF-E2, Nrf-2, Pax-2, Pou5f1, TCF11::MafG, p300 |  | *NRXN1* | intronic |
| 50976865 | rs66971536 |  | ESC |  |  | Pou5f1, SIX5, Znf143 |  | *NRXN1* | intronic |
| 50979204 | rs35296842 |  | BRST, BRN, PANC |  |  | Maf, NF-kappaB |  | *NRXN1* | intronic |
| 50981010 | rs7571051 |  |  |  |  | Mef2, Pou3f2 |  | *NRXN1* | intronic |
| 50982262 | rs10490164 |  |  |  |  | GR |  | *NRXN1* | intronic |
| 50984688 | rs17573413 |  |  |  |  | Cdx2, Irf, ZBRK1 |  | *NRXN1* | intronic |
| 50986740 | rs11356752 |  |  |  |  | CIZ, Foxp1, NF-AT1, STAT, p300 |  | *NRXN1* | intronic |
| 50987855 | rs7574611 |  |  | MUS |  | AP-4, Ascl2, HEN1, LBP-1 | 1 hit | *NRXN1* | intronic |
| 50989672 | rs13031157 |  |  |  |  |  | 1 hit | *NRXN1* | intronic |
| 50990502 | rs17573587 |  |  | MUS |  |  |  | *NRXN1* | intronic |
| 50991772 | rs10490163 |  |  | SKIN, MUS |  | CCNT2, Evi-1, GATA, HDAC2, HMGN3, PLZF, ZEB1 |  | *NRXN1* | intronic |
| 50991932 | rs918138 |  | PANC | SKIN, MUS |  | GATA, Maf |  | *NRXN1* | intronic |
| 50992226 | rs918139 |  | BRN, PANC |  |  | Foxp1, GR, Pou2f2, Pou3f3 |  | *NRXN1* | intronic |
| 50992517 | rs147083758 |  | BRN, PANC |  |  | Arid3a, Hoxa10, Hoxb13, Mef2, Nkx2, PLZF, TATA |  | *NRXN1* | intronic |
| 50993079 | rs6718913 |  | ESDR, PANC |  |  | DMRT7, HP1-site-factor |  | *NRXN1* | intronic |
| 50995719 | rs6714295 |  | MUS, GI | ESDR, KID, MUS, LNG |  | CTCF, Hsf, Irf, PRDM1, Pax-5, Rad21, p300 |  | *NRXN1* | intronic |
| 50999569 | rs7599370 |  | ESDR, ESC, IPSC, BRN, PANC |  |  | Pax-5 |  | *NRXN1* | intronic |
| 51002858 | rs17514717 |  | ESC, IPSC, BLD, PANC |  |  |  |  | *NRXN1* | intronic |
| 51004094 | rs17514766 | BLD | ESC, IPSC, BLD, BRN, VAS | IPSC | SP1 | PLZF |  | *NRXN1* | intronic |
| 51004722 | rs34684671 |  | IPSC, BLD |  |  | RFX5, RXRA, TR4 |  | *NRXN1* | intronic |
| 51005739 | rs68180403 |  | IPSC |  |  | Cdx2, Foxp1, HNF1, Hoxa9, Hoxb9, Pou2f2 |  | *NRXN1* | intronic |
| 51010629 | rs7594170 |  | ESC, BRN, PANC |  |  | Bcl6b, Irf, Pax-5, ZEB1 | 1 hit | *NRXN1* | intronic |
| 51017499 | rs12990172 |  |  |  |  | Hand1, Myf, Pax-4, RP58 |  | *NRXN1* | intronic |
| 51021506 | rs35397701 |  | BRN |  |  | Arid3a, Dbx1, Hoxa10, Hoxa3, Hoxa5, Hoxa7, Hoxa9, Hoxb4, Hoxc6, Hoxd10, Hoxd8, Isl2, Lhx3, Ncx, Nkx6-1, Pax7, Pou2f2, Pou3f2, Pou3f4, Prrx1, Prrx2, Sox, Zfp105 |  | *NRXN1* | intronic |
| 51021592 | rs58624935 |  | BRN |  |  | Nkx2, RORalpha1, Sox |  | *NRXN1* | intronic |
| 51022746 | rs17574342 | BRN | BRN, PANC |  |  | DMRT2, DMRT7, Foxk1, Foxo, Homez, Sox |  | *NRXN1* | intronic |
| 51023367 | rs7557525 | BRN | BRN, PANC |  |  | HNF4, Maf, Nkx2, PPAR, PU.1, VDR |  | *NRXN1* | intronic |
| 51027340 | rs13023341 | BRN | BLD, BRN |  |  |  |  | *NRXN1* | intronic |
| 51028694 | rs67661616 | BLD, BRN | ESDR, BLD, BRN, MUS | ESDR |  | AIRE, CTCF, TLX1::NFIC |  | *NRXN1* | 5'-UTR |
| 51070748 | rs12993750 |  | BLD |  |  | AP-1, HNF1, Hoxa5 |  | 38kb 5' of *NRXN1* | intronic |
| 51077100 | rs2163018 |  | ESDR |  |  | Mef2, RXRA |  | 45kb 5' of *NRXN1* | intronic |

**Supplementary Table 4B.** HaploReg annotations for the significant *cis*-regulatory variants detected in *CYP2A7* on chromosome 19

| Pos (hg38) | SNP ID | Promoter histone marks | Enhancer histone marks | DNA source | Proteins bound | Motifs changed | Selected eQTL hits | RefSeq gene | dbSNP location |
| --- | --- | --- | --- | --- | --- | --- | --- | --- | --- |
| 40999066 | rs10424026 |  |  |  |  | CEBPB, p300 |  | *CYP2B6* | intronic |
| 41000286 | rs3786547 | LNG | ESDR, SKIN, LIV, GI, BRST | GI, LNG, BRST | GR | RXR::LXR, SZF1-1 |  | *CYP2B6* | intronic |
| 41000760 | rs2200237 |  | LIV, GI | GI |  | Egr-1, SETDB1, Znf143 |  | *CYP2B6* | intronic |
| 41002537 | rs10500282 |  |  |  |  | AIRE, Foxa, HDAC2, Sox, Zfp105, p300 |  | *CYP2B6* | intronic |
| 41003533 | rs10403955 |  |  | ESDR |  |  |  | *CYP2B6* | intronic |
| 41010277 | rs2306606 |  |  |  |  | AIRE |  | *CYP2B6* | intronic |
| 41010444 | rs34906003 |  |  |  |  | Nrf-2, TCF11::MafG, ZID |  | *CYP2B6* | intronic |
| 41011041 | rs34830389 |  |  |  |  | Bbx, Foxj1, Hbp1, Pitx2 |  | *CYP2B6* | intronic |
| 41014939 | rs11673270 |  | BRST |  |  | EWSR1-FLI1, HDAC2, Hoxa9, TATA, p300 |  | *CYP2B6* | intronic |
| 41024741 | rs10401226 |  |  | LNG |  | CEBPB, E2A, Ets, Foxo, Myf, NF-AT1, TAL1, VDR |  | 6.3kb 3' of *CYP2B6* |  |
| 41025307 | rs3895941 |  | IPSC |  |  | Ncx, STAT |  | 6.9kb 3' of *CYP2B6* |  |
| 41025617 | rs11670865 |  | IPSC, BRST |  |  | CTCF, HNF4, Rad21, SMC3 |  | 7.2kb 3' of *CYP2B6* |  |
| 41025751 | rs7249735 | ESC | IPSC, ESC, BRST, PLCNT, LNG |  |  | CDP |  | 7.4kb 3' of *CYP2B6* |  |
| 41025800 | rs3745275 | ESC | IPSC, ESC, BRST, PLCNT, LNG |  |  | CCNT2, Irf, SP1, STAT, Zfp281 |  | 7.4kb 3' of *CYP2B6* |  |
| 41025954 | rs7254767 | IPSC | ESDR, ESC, IPSC, BRST, LIV, GI, PLCNT, PANC | ESC, IPSC |  | Hic1 |  | 7.6kb 3' of *CYP2B6* |  |
| 41026010 | rs3745277 | IPSC | ESDR, ESC, IPSC, BRST, LIV, GI, PLCNT, PANC | ESC, IPSC | CTCF, RAD21 | ATF3, Egr-1, HES1, Myc, Nrf1, SETDB1, p300 |  | 7.6kb 3' of *CYP2B6* |  |
| 41026221 | rs7255146 | IPSC | ESC, ESDR, BRST, PLCNT, PANC | IPSC, | CTCF | Hsf, p300 |  | 7.8kb 3' of *CYP2B6* |  |
| 41026236 | rs71742418 | IPSC | ESC, ESDR, BRST, PLCNT, PANC | IPSC |  | AP-1, Pax-5, p300 |  | 7.8kb 3' of *CYP2B6* |  |
| 41026374 | rs11879790 |  | ESC, IPSC |  |  | EWSR1-FLI1, HDAC2, MZF1::1-4, PU.1, SP1, STAT, TATA, VDR, Znf143, p300 |  | 8kb 3' of *CYP2B6* |  |
| 41026749 | rs10419321 |  |  |  |  | HNF4, RAR, TATA |  | 8.4kb 3' of *CYP2B6* |  |
| 41026833 | rs10420699 |  |  |  |  | Irf, Nanog, Pax-5, p300 |  | 8.4kb 3' of *CYP2B6* |  |
| 41026980 | rs10424716 |  |  |  |  | Ets, KAP1, MAZ, PU.1, Znf143 |  | 8.6kb 3' of *CYP2B6* |  |
| 41027104 | rs10426482 |  |  |  |  | CACD, PEBP |  | 8.7kb 3' of *CYP2B6* |  |
| 41027123 | rs10424952 |  |  |  |  | Rad21 |  | 8.7kb 3' of *CYP2B6* |  |
| 41027144 | rs8192785 |  |  |  |  | CHD2, SRF |  | 8.7kb 3' of *CYP2B6* |  |
| 41027182 | rs10426686 |  |  |  |  | ATF3 |  | 8.8kb 3' of *CYP2B6* |  |
| 41027319 | rs10425769 |  |  | IPSC |  |  |  | 8.9kb 3' of *CYP2B6* |  |
| 41027351 | rs11673685 |  |  |  |  | CTCF, Egr-1, Zfp410 |  | 9kb 3' of *CYP2B6* |  |
| 41028085 | rs8103372 |  | GI |  |  | RREB-1 |  | 9.7kb 3' of *CYP2B6* |  |
| 41028164 | rs8102557 |  |  |  |  | MIF-1, ZEB1 |  | 9.8kb 3' of *CYP2B6* |  |
| 41028166 | rs8102771 |  |  |  |  | ZEB1 |  | 9.8kb 3' of *CYP2B6* |  |
| 41029036 | rs28671673 |  |  |  |  | BDP1, LUN-1, Pax-4, RREB-1, UF1H3BETA, VDR |  | 11kb 3' of *CYP2B6* |  |
| 41029071 | rs10413524 |  |  |  |  | AP-2, BATF, EBF, GATA, GR, PTF1-beta, SP2 |  | 11kb 3' of *CYP2B6* |  |
| 41029190 | rs10415473 |  |  |  |  |  |  | 11kb 3' of *CYP2B6* |  |
| 41029385 | rs11671743 |  |  |  |  | TCF12 |  | 11kb 3' of *CYP2B6* |  |
| 41029443 | rs11667928 |  |  |  |  | HEN1 |  | 11kb 3' of *CYP2B6* |  |
| 41029515 | rs11667951 |  | PLCNT |  |  | GCNF, Hic1, Rad21 |  | 11kb 3' of *CYP2B6* |  |
| 41030049 | rs16974878 |  | LIV, PLCNT, OVRY |  |  | HES1, SEF-1 |  | 12kb 3' of *CYP2B6* |  |
| 41030212 | rs10421597 |  |  | OVRY |  | Ik-1, ZEB1 |  | 12kb 3' of *CYP2B6* |  |
| 41030242 | rs10422829 |  |  | OVRY |  | ERalpha-a, Egr-1, GR, PPAR, RAR, RORalpha1, RXRA, VDR |  | 12kb 3' of *CYP2B6* |  |
| 41030332 | rs10421834 |  |  |  |  | BCL, Gcm1 |  | 12kb 3' of *CYP2B6* |  |
| 41030511 | rs10422282 |  |  | OVRY |  | AP-1, ATF4, HEY1, SIX5, TATA, Znf143 |  | 12kb 3' of *CYP2B6* |  |
| 41030598 | rs10422151 |  |  | LNG |  | Irx, Nkx3 |  | 12kb 3' of *CYP2B6* |  |
| 41030642 | rs10423743 |  |  | ESC, IPSC |  |  |  | 12kb 3' of *CYP2B6* |  |
| 41030737 | rs10422729 |  |  | ESC, IPSC, PLCNT, OVRY | CTCF, RAD21 | EBF, EWSR1-FLI1, Irf, MZF1::1-4, PRDM1, PU.1, RXRA, SP1, STAT, UF1H3BETA, VDR, Zfp281 |  | 12kb 3' of *CYP2B6* |  |
| 41031056 | rs10402037 |  |  | ESDR |  | DMRT1, DMRT4, Irf, Pax-8, Pou2f2 |  | 13kb 3' of *CYP2B6* |  |
| 41031140 | rs10402611 |  |  |  |  | AP-1 |  | 13kb 3' of *CYP2B6* |  |
| 41031145 | rs10402070 |  |  |  |  | AP-1 |  | 13kb 3' of *CYP2B6* |  |
| 41031421 | rs10403268 |  |  |  |  | ATF3 |  | 13kb 3' of *CYP2B6* |  |
| 41031543 | rs10403140 |  |  |  |  | Pax-5 |  | 13kb 3' of *CYP2B6* |  |
| 41031605 | rs10403330 |  |  |  |  | Hand1, Smad3, Smad |  | 13kb 3' of *CYP2B6* |  |
| 41031945 | rs10410867 |  |  | ESDR, PLCNT, BRST |  | Maf, PU.1, STAT |  | 14kb 3' of *CYP2B6* |  |
| 41031963 | rs10409701 |  |  | ESDR, PLCNT | P300 | FAC1, HMG-IY, STAT, Sox |  | 14kb 3' of *CYP2B6* |  |
| 41032087 | rs10409738 |  |  | ESDR, BRST, SKIN, PLCNT, OVRY | STAT3, ERALPHA_A, FOXA1, GATA3, P300 |  |  | 14kb 3' of *CYP2B6* |  |
| 41032154 | rs10409585 |  |  | ESDR, BRST, PLCNT | STAT3, ERALPHA_A, FOXA1, P300 | BCL, Irf, Pax-4, Pax-5, STAT |  | *14kb 3' of CYP2B6* |  |
| 41032203 | rs10410347 |  |  | ESDR, BRST, PLCNT | STAT3, ERALPHA_A, FOXA1, P300 | Mef2 |  | 14kb 3' of *CYP2B6* |  |
| 41032833 | rs1080235 |  |  |  |  | Nkx3 |  | 14kb 3' of *CYP2B6* |  |
| 41032836 | rs1080234 |  |  |  |  |  |  | 14kb 3' of *CYP2B6* |  |
| 41033120 | rs1807967 |  | ESC, BRST, BLD, PLCNT |  |  | AP-1, ATF3, Egr-1 |  | 15kb 3' of *CYP2B6* |  |
| 41033227 | rs10417678 |  |  |  |  |  |  | 15kb 3' of *CYP2B6* |  |
| 41033302 | rs142408152 |  | ESC, BLD, PLCNT |  |  | AP-1, COMP1, CTCFL, E2A, NF-AT1, STAT, TAL1, TCF12, YY1 |  | 15kb 3' of *CYP2B6* |  |
| 41033352 | rs10419098 |  |  | ESDR |  | RXRA, Zbtb3 |  | 15kb 3' of *CYP2B6* |  |
| 41033654 | rs10418063 |  |  | ESC, BLD |  | Hsf, TATA |  | 15kb 3' of *CYP2B6* |  |
| 41033713 | rs10418426 |  |  |  |  | E2A, Ik-1 |  | 15kb 3' of *CYP2B6* |  |
| 41033818 | rs10418657 |  |  |  |  | ERalpha-a, Eomes, Esr2, Mrg, SIX5, TBX5, Tgif1, p300 |  | 15kb 3' of *CYP2B6* |  |
| 41033826 | rs10418473 |  |  |  |  | p300 |  | 15kb 3' of *CYP2B6* |  |
| 41034085 | rs10425932 |  |  |  |  | SIX5 |  | 16kb 3' of *CYP2B6* |  |

Note: Rs373754258 was not found in 1000 Genomes Phase 1 data, thus not annotated by HaploReg.

**Supplementary Table 4C.** HaploReg annotations for the significant *cis*-regulatory variants detected in *EGLN2* on chromosome 19

| Pos (hg38) | SNP ID | Promoter histone marks | Enhancer histone marks | DNA Source | Proteins bound | Motifs changed | Selected eQTL hits | RefSeq gene | SNP location |
| --- | --- | --- | --- | --- | --- | --- | --- | --- | --- |
| 40739215 | rs117032961 |  | SKIN, BRN, GI, PLCNT | SKIN, GI, LNG, BRST |  | CTCF, GATA, Myf, Pax-5, RXRA, Rad21, SMC3, TAL1 |  | *ITPKC* | intronic |
| 40740333 | rs139556022 | FAT, STRM, BRN, SKIN | ESDR, IPSC, SKIN, BRN, GI, MUS, PLCNT, OVRY, HRT, LNG | SKIN, ADRL, GI, LNG, BRST |  | Ets, Myf, Pax-5, VDR |  | *ITPKC* | 3'-UTR |
| 40740983 | rs117499206 |  | FAT, STRM, SKIN, BRN, GI, MUS, PLCNT, HRT | SKIN, LNG, MUS, PLCNT, GI, OVRY, LNG, BRST | POL2 | AP-1 |  | *C19orf54* | 3'-UTR |
| 40741002 | rs62621363 |  | FAT, STRM, SKIN, BRN, GI, MUS, PLCNT, HRT | SKIN, LNG, MUS, GI, OVRY, BRST, LNG | POL2 | Mef2 |  | *C19orf54* | 3'-UTR |
| 40742011 | rs201038736 |  | BRST, STRM, GI, ADRL, MUS, PLCNT, BLD | ESDR, BRST, ADRL, PLCNT, MUS, LNG, BLD | CTCF, POL2 | BRCA1 |  | *C19orf54* |  |
| 40743318 | rs142449067 |  | PLCNT |  |  | BCL, ERalpha-a, Irf, SP1, ZBRK1 |  | *C19orf54* | intronic |
| 40743511 | rs145469712 |  | PLCNT |  |  |  |  | *C19orf54* | intronic |
| 40746167 | rs4803362 |  | ESDR, ADRL, GI, PLCNT, MUS, LNG, LIV, BONE | ESDR, PLCNT, MUS |  | AP-2, SRF |  | *C19orf54* | intronic |
| 40747170 | rs117354824 |  |  | MUS |  | NRSF, PPAR, RREB-1, UF1H3BETA, YY1, ZBTB7A, ZNF219, Zfp281, Zfp740 |  | *C19orf54* | intronic |
| 40747583 | rs151246387 |  |  | IPSC, OVRY |  | ERalpha-a, Mtf1, YY1, Zbtb3, Znf143, p300 |  | *C19orf54* | intronic |
| 40749863 | rs4802088 | ESC, ESDR, IPSC, FAT, STRM, BRST, BLD, MUS, BRN, SKIN, LIV, GI, ADRL, PANC, PLCNT, THYM, HRT, LNG, CRVX, BONE | ESDR, LNG, BRST, BLD, BRN, HRT, KID, MUS, GI, PLCNT, SPLN, VAS | ESDR, BLD, SKIN, ADRL, HRT, GI, MUS, PLCNT, OVRY, CRVX | POL2, E2F6 | Rad21 |  | *C19orf54* | 5'-UTR |
| 40750587 | rs117375307 | ESC, ESDR, LNG, IPSC, FAT, STRM, BRST, BLD, MUS, BRN, SKIN, VAS, LIV, GI, ADRL, HRT, KID, PANC, PLCNT, THYM, OVRY, SPLN, CRVX, BONE | BLD | ESC, ESDR, LNG, IPSC, BRST, BLD, SKIN, ADRL, BRN, HRT, GI, KID, MUS, PLCNT, THYM, OVRY, PANC, CRVX, LIV, VAS | GR, USF1, NFKB, POL2, CMYC, ELF1, GABP, MAX, NFE2, SIN3AK20, SRF, TBP, USF2, YY1, ZBTB33, ATF3, BRCA1, CEBPB, E2F6, GTF2F1, HAE2F1, MXI1, POL2S2, RFX5, TAF1, CHD2, P300, EGR1, STAT2, STAT3 | ELF1, MOVO-B, NRSF, SZF1-1, Sin3Ak-20 |  | 265bp 5' of *SNRPA* |  |
| 40752476 | rs112046926 | ESC, ESDR, IPSC, FAT, STRM, BRST, BLD, MUS, BRN, SKIN, LIV, GI, ADRL, KID, PANC, LNG, LCNT, OVRY, HRT, THYM, CRVX, VAS, BONE | ESDR, BLD, BRN, HRT, LNG, PLCNT, MUS, SPLN | ESDR, BLD, PLCNT, OVRY, MUS, GI, LNG, LIV, SKIN | POL2 | Ets, LBP-1, Pax-5, Zfx |  | *SNRPA* | intronic |
| 40753017 | rs79570674 | ESDR, IPSC, FAT, BRST, BLD, SKIN, GI, CRVX, LIV, VAS, BRN, BONE | ESDR, LNG, ESC, STRM, BRST, BLD, MUS, BRN, LIV, GI, ADRL, PLCNT, THYM, HRT, SPLN, SKIN | BLD, GI |  | NRSF |  | *SNRPA* | intronic |
| 40754818 | rs80208490 | ESDR, BLD, LIV, GI, PANC, BONE | ESDR, LNG, IPSC, FAT, ESC, STRM, BRST, BLD, MUS, BRN, SKIN, GI, ADRL, HRT, KID, PANC, THYM, OVRY, SPLN | ESDR, BLD, GI, PLCNT, LNG, LIV, SKIN | POL2, AP2ALPHA, AP2GAMMA, HNF4A, HNF4G | AP-4, CTCF, ELF1, HEN1, LBP-1, Nanog, RP58 |  | *SNRPA* | intronic |
| 40760937 | rs144761778 |  | BLD, ADRL, VAS | MUS |  | DMRT7, Foxp1, PPAR, Sox, Zfp105, p300 |  | *SNRPA* | intronic |
| 40763460 | rs4803365 |  | ESDR, BLD, BRN, SKIN | LNG |  | AIRE, CEBPB, Hltf, Osf2, RREB-1, SRF, Sox |  | *SNRPA* | intronic |
| 40765629 | rs112889062 |  | BRST, BLD, PLCNT, THYM, SPLN, VAS | BLD, THYM, VAS |  | YY1 |  | 236bp 3' of *SNRPA* |  |
| 40765920 | rs144374087 |  | BLD, PLCNT, THYM, SPLN, VAS | BLD, THYM |  |  |  | 527bp 3' of *SNRPA* |  |
| 40767539 | rs200795716 |  |  |  |  | Foxl1, Foxp1, Pou1f1, Pou2f2, TATA |  | 2.1kb 3' of *SNRPA* |  |
| 40767717 | rs144243973 |  |  |  |  | ERalpha-a, RXRA, T3R |  | 2.3kb 3' of *SNRPA* |  |
| 40768066 | rs139707534 |  |  |  |  | AhR::Arnt, Arnt, HNF4, Pax-8 |  | 2.7kb 3' of *SNRPA* |  |
| 40769820 | rs191883259 |  | BLD |  |  | LBP-1, SZF1-1, Spz1, ZBTB33 |  | 4.4kb 3' of *SNRPA* |  |
| 40770150 | rs116972110 |  | BLD | IPSC |  | Zbtb3 |  | 4.8kb 3' of *SNRPA* |  |
| 40772588 | rs146641468 |  |  |  |  | HNF4, Maf, Zbtb3 |  | 2.6kb 5' of *MIA* |  |
| 40773237 | rs141788519 |  | BLD |  |  |  |  | 1.9kb 5' of *MIA* |  |
| 40774143 | rs146221490 |  | BRST, BLD, SKIN, PLCNT | PLCNT, OVRY |  | EBF, GCNF, Rad21, SREBP, Zic |  | 1kb 5' of *MIA* |  |
| 40774645 | rs2233149 |  | ESDR, BRST, BLD, STRM, SKIN, PLCNT |  |  | ATF2, Barx1, Barx2, Bsx, CEBPB, CEBPG, Dbx1, Dbx2, Dlx2, Dlx3, En-1, Foxj1, Hlx1, Hoxa10, Hoxa7, Hoxb4, Hoxc6, Hoxd8, Msx-1, Ncx, Nkx3, Nkx6-1, Pax-6, Pou2f2, Pou3f2, Pou3f4, Sox, TATA |  | 530bp 5' of *MIA* |  |
| 40776467 | rs117248593 | BRST, STRM | ESDR, BLD, BRN, SKIN | BLD, BRN, OVRY |  | AP-4, Ascl2, E2A, HEN1, LBP-1, Lmo2-complex, NRSF, Pou2f2 |  | *MIA* | intronic |
| 40777562 | rs79016062 | ESC, ESDR, LNG, IPSC, FAT,STRM, BRST, BLD, MUS, BRN, SKIN, VAS, LIV, GI, ADRL,HRT, KID, PANC, PLCNT, THYM, OVRY, CRVX, BONE |  | ESC, ESDR, LNG, IPSC, BRST, BLD, SKIN, ADRL, BRN, HRT, GI, KID, MUS, PLCNT, THYM, OVRY, PANC, CRVX, LIV, VAS | POL2, RFX5, ZNF143, NFKB, TBP, BRCA1, CEBPB, IRF3, USF2, CFOS, GTF2F1, NFYA, NFYB | DBP, NRSF |  | *MIA-RAB4B* | intronic |
| 40778492 | rs141715458 | ESC, ESDR, LNG, IPSC, FAT, STRM, BRST, BLD, MUS, BRN, SKIN, VAS, LIV, GI, ADRL, HRT, KID, PANC, PLCNT, THYM, OVRY, SPLN, CRVX, BONE |  | ESC, ESDR, LNG, IPSC, BRST, BLD, SKIN, ADRL, BRN, HRT, GI, KID, MUS, PLCNT, THYM, OVRY, PANC, CRVX, LIV, VAS | POL2, EGR1, SP1, AP2ALPHA, AP2GAMMA, BHLHE40, IRF1, POL24H8 | ATF3, Hand1, Pax-4 |  | *RAB4B* | intronic |
| 40778641 | rs146247868 | ESC, ESDR, LNG, IPSC, FAT, STRM, BRST, BLD, MUS, BRN, SKIN, VAS, LIV, GI, ADRL, HRT, KID, PANC, PLCNT, THYM, OVRY, SPLN, CRVX, BONE |  | ESC, ESDR, LNG, IPSC, BRST, BLD, SKIN, ADRL, BRN, HRT, GI, KID, MUS, PLCNT, THYM, OVRY, PANC, CRVX, LIV, VAS | EGR1, SP1, POL2, AP2ALPHA, AP2GAMMA, IRF1 | BDP1, PLAG1 |  | *RAB4B* | intronic |
| 40779968 | rs4803367 | BLD | ESC, ESDR, IPSC, FAT, BLD, SKIN, BRN, ADRL, PLCNT, SPLN | BLD |  | E2F, YY1 |  | *RAB4B* | intronic |
| 40781668 | rs142449117 |  | ESC, PLCNT, GI, BLD |  |  |  |  | *RAB4B* | intronic |
| 40789005 | rs113557544 |  |  |  |  | FXR, RAR, RXRA, STAT |  | *RAB4B* | intronic |
| 40790574 | rs113336186 |  | PLCNT |  |  |  |  | *RAB4B* | intronic |
| 40793856 | rs111684981 |  | BLD, PLCNT |  |  | AIRE |  | *RAB4B* | intronic |
| 40793961 | rs149755483 |  | PLCNT |  |  | AIRE, FAC1, Foxa, Foxp1, Irf, Nanog, Pax-4, RREB-1, Sox, Zfp105, p300 |  | *RAB4B* | intronic |
| 40795164 | rs7251781 |  | PLCNT |  |  | ZID |  | *RAB4B* | intronic |
| 40796235 | rs149397079 |  | IPSC, PLCNT, THYM | PLCNT |  | ZID |  | *RAB4B* | intronic |
| 40796470 | rs117947334 |  | BLD, PLCNT | ESDR, PLCNT, BLD |  | CDP, TAL1 |  | *RAB4B* | intronic |
| 40796596 | rs45600135 |  | IPSC, BLD, SKIN, PLCNT | BLD, PLCNT, OVRY |  | CTCF, Nanog |  | *RAB4B* | 3'-UTR |
| 40799625 | rs34406232 | ESC, ESDR, LNG, IPSC, FAT, STRM, BRST, BLD, MUS, BRN, SKIN, VAS, LIV, GI, ADRL, HRT, KID, PANC, PLCNT, THYM, OVRY, SPLN, CRVX, BONE |  | ESC, ESDR, IPSC, BRST, BLD, SKIN, HRT, GI, LNG, MUS, PLCNT, OVRY, LIV, BRN | POL2, ELF1, POL24H8, HEY1, ZBTB7A, ZNF263 | Evi-1 |  | *EGLN2* | 5'-UTR |
| 40803245 | rs117391664 | FAT, BLD, GI, HRT, MUS, LIV, VAS | ESC, ESDR, LNG, IPSC, STRM, BRST, BLD, BRN, LIV, GI, HRT, MUS, PLCNT, THYM, OVRY, PANC, CRVX, SKIN | BLD, HRT, GI, OVRY, PANC |  | Hand1, Mrg1::Hoxa9 |  | *EGLN2* | intronic |
| 40803685 | rs117851058 | BLD, FAT, GI, HRT, LNG, LIV, VAS | ESC, ESDR, LNG, IPSC, FAT, STRM, BRST, BLD, MUS, BRN, SKIN, LIV, GI, HRT, PLCNT, THYM, OVRY, PANC, SPLN, CRVX, BONE | ESDR, ESC, IPSC, BLD, GI, THYM, OVRY, PANC, LIV | POL2 | E2A, HEN1 |  | *EGLN2* | intronic |
| 40804983 | rs117821847 | BLD, BRN | ESC, ESDR, LNG, IPSC, FAT, STRM, BRST, BLD, MUS, BRN, SKIN, LIV, GI, ADRL, HRT, PLCNT, THYM, OVRY, PANC, SPLN, VAS | BLD, SKIN, PANC, MUS, LNG | CTCF, POL2, POL24H8, CMYC | PEBP |  | *EGLN2* | intronic |
| 40806017 | rs117139969 | BLD, RN, MUS | ESC, ESDR, LNG, IPSC, STRM, BRST, BLD, BRN, SKIN, FAT, LIV, GI, ADRL, HRT, KID, PANC, MUS, PLCNT, THYM, SPLN, VAS | IPSC |  |  |  | *EGLN2* | intronic |
| 40807356 | rs144553930 | ESDR, BLD, BRN | ESC, ESDR, LNG, IPSC, FAT, BRST, BLD, STRM, MUS, BRN, SKIN, LIV, GI, ADRL, PANC, PLCNT, THYM, HRT, SPLN, VAS, BONE | ESC, BLD, BRN, PLCNT | POL24H8 | AP-1, ATF3, VDR |  | *EGLN2* | intronic |
| 40809421 | rs149812270 |  | IPSC, ESC, BRST, BLD, SKIN, FAT, LIV, BRN, GI, MUS, PLCNT, HRT, LNG, PANC, SPLN | BLD, PLCNT, OVRY | POL2, POL24H8 | ZBTB33 |  | 979bp 3' of *EGLN2* |  |
| 40813721 | rs193122850 |  | ESC, ESDR, LNG, BRST, BRN, SKIN, GI, ADRL, HRT, KID, MUS, PLCNT, OVRY, BLD | BLD, SKIN |  | LUN-1 |  | 5.3kb 3' of *EGLN2* | missense |

**References:**

[1] J. Wessel, S.M. McDonald, D.A. Hinds, R.P. Stokowski, H.S. Javitz, M. Kennemer, R. Krasnow, W. Dirks, J. Hardin, S.J. Pitts, M. Michel, L. Jack, D.G. Ballinger, J.B. McClure, G.E. Swan, and A.W. Bergen, Resequencing of nicotinic acetylcholine receptor genes and association of common and rare variants with the Fagerstrom test for nicotine dependence. Neuropsychopharmacology 35 (2010) 2392-402.

[2] J. Nussbaum, Q. Xu, T.J. Payne, J.Z. Ma, W. Huang, J. Gelernter, and M.D. Li, Significant association of the neurexin-1 gene (NRXN1) with nicotine dependence in European- and African-American smokers. Hum Mol Genet 17 (2008) 1569-77.

[3] N. Sato, S. Kageyama, R. Chen, M. Suzuki, F. Tanioka, T. Kamo, K. Shinmura, A. Nozawa, and H. Sugimura, Association between neurexin 1 (NRXN1) polymorphisms and the smoking behavior of elderly Japanese. Psychiatr Genet 20 (2010) 135-6.

[4] J. Yang, S. Wang, Z. Yang, C.A. Hodgkinson, P. Iarikova, J.Z. Ma, T.J. Payne, D. Goldman, and M.D. Li, The contribution of rare and common variants in 30 genes to risk nicotine dependence. Mol Psychiatry 20 (2015) 1467-78.

[5] L.J. Bierut, P.A. Madden, N. Breslau, E.O. Johnson, D. Hatsukami, O.F. Pomerleau, G.E. Swan, J. Rutter, S. Bertelsen, L. Fox, D. Fugman, A.M. Goate, A.L. Hinrichs, K. Konvicka, N.G. Martin, G.W. Montgomery, N.L. Saccone, S.F. Saccone, J.C. Wang, G.A. Chase, J.P. Rice, and D.G. Ballinger, Novel genes identified in a high-density genome wide association study for nicotine dependence. Hum Mol Genet 16 (2007) 24-35.

[6] S.F. Saccone, A.L. Hinrichs, N.L. Saccone, G.A. Chase, K. Konvicka, P.A. Madden, N. Breslau, E.O. Johnson, D. Hatsukami, O. Pomerleau, G.E. Swan, A.M. Goate, J. Rutter, S. Bertelsen, L. Fox, D. Fugman, N.G. Martin, G.W. Montgomery, J.C. Wang, D.G. Ballinger, J.P. Rice, and L.J. Bierut, Cholinergic nicotinic receptor genes implicated in a nicotine dependence association study targeting 348 candidate genes with 3713 SNPs. Hum Mol Genet 16 (2007) 36-49.

[7] A. Agrawal, M.L. Pergadia, S.F. Saccone, A.L. Hinrichs, C.N. Lessov-Schlaggar, N.L. Saccone, R.J. Neuman, N. Breslau, E. Johnson, D. Hatsukami, G.W. Montgomery, A.C. Heath, N.G. Martin, A.M. Goate, J.P. Rice, L.J. Bierut, and P.A.F. Madden, Gamma-aminobutyric acid receptor genes and nicotine dependence: evidence for association from a case-control study. Addiction 103 (2008) 1027-1038.

[8] A. Agrawal, M.L. Pergadia, S. Balasubramanian, S.F. Saccone, A.L. Hinrichs, N.L. Saccone, N. Breslau, E.O. Johnson, D. Hatsukami, N.G. Martin, G.W. Montgomery, A.M. Goate, J.P. Rice, L.J. Bierut, and P.A. Madden, Further evidence for an association between the gamma-aminobutyric acid receptor A, subunit 4 genes on chromosome 4 and Fagerstrom Test for Nicotine Dependence. Addiction 104 (2009) 471-7.

[9] W. Huang, J.Z. Ma, T.J. Payne, J. Beuten, R.T. Dupont, and M.D. Li, Significant association of DRD1 with nicotine dependence. Hum Genet 123 (2008) 133-40.

[10] M.R. Munafo, K.M. Elliot, M.F. Murphy, R.T. Walton, and E.C. Johnstone, Association of the mu-opioid receptor gene with smoking cessation. Pharmacogenomics J 7 (2007) 353-61.

[11] R.A. Grucza, E.O. Johnson, R.F. Krueger, N. Breslau, N.L. Saccone, L.S. Chen, J. Derringer, A. Agrawal, M. Lynskey, and L.J. Bierut, Incorporating age at onset of smoking into genetic models for nicotine dependence: evidence for interaction with multiple genes. Addict Biol 15 (2010) 346-57.

[12] Y. Yu, C. Panhuysen, H.R. Kranzler, V. Hesselbrock, B. Rounsaville, R. Weiss, K. Brady, L.A. Farrer, and J. Gelernter, Intronic variants in the dopa decarboxylase (DDC) gene are associated with smoking behavior in European-Americans and African-Americans. Hum Mol Genet 15 (2006) 2192-9.

[13] J.Z. Ma, J. Beuten, T.J. Payne, R.T. Dupont, R.C. Elston, and M.D. Li, Haplotype analysis indicates an association between the DOPA decarboxylase (DDC) gene and nicotine dependence. Hum Mol Genet 14 (2005) 1691-8.

[14] J. Gelernter, H.R. Kranzler, R. Sherva, L. Almasy, A.I. Herman, R. Koesterer, H. Zhao, and L.A. Farrer, Genome-wide association study of nicotine dependence in American populations: identification of novel risk loci in both African-Americans and European-Americans. Biol Psychiatry 77 (2015) 493-503.

[15] T.E. Thorgeirsson, D.F. Gudbjartsson, I. Surakka, J.M. Vink, N. Amin, F. Geller, P. Sulem, T. Rafnar, T. Esko, S. Walter, C. Gieger, R. Rawal, M. Mangino, I. Prokopenko, R. Magi, K. Keskitalo, I.H. Gudjonsdottir, S. Gretarsdottir, H. Stefansson, J.R. Thompson, Y.S. Aulchenko, M. Nelis, K.K. Aben, M. den Heijer, A. Dirksen, H. Ashraf, N. Soranzo, A.M. Valdes, C. Steves, A.G. Uitterlinden, A. Hofman, A. Tonjes, P. Kovacs, J.J. Hottenga, G. Willemsen, N. Vogelzangs, A. Doring, N. Dahmen, B. Nitz, M.L. Pergadia, B. Saez, V. De Diego, V. Lezcano, M.D. Garcia-Prats, S. Ripatti, M. Perola, J. Kettunen, A.L. Hartikainen, A. Pouta, J. Laitinen, M. Isohanni, S. Huei-Yi, M. Allen, M. Krestyaninova, A.S. Hall, G.T. Jones, A.M. van Rij, T. Mueller, B. Dieplinger, M. Haltmayer, S. Jonsson, S.E. Matthiasson, H. Oskarsson, T. Tyrfingsson, L.A. Kiemeney, J.I. Mayordomo, J.S. Lindholt, J.H. Pedersen, W.A. Franklin, H. Wolf, G.W. Montgomery, A.C. Heath, N.G. Martin, P.A. Madden, I. Giegling, D. Rujescu, M.R. Jarvelin, V. Salomaa, M. Stumvoll, T.D. Spector, H.E. Wichmann, A. Metspalu, N.J. Samani, B.W. Penninx, B.A. Oostra, D.I. Boomsma, H. Tiemeier, C.M. van Duijn, J. Kaprio, J.R. Gulcher, M.I. McCarthy, L. Peltonen, U. Thorsteinsdottir, and K. Stefansson, Sequence variants at CHRNB3-CHRNA6 and CYP2A6 affect smoking behavior. Nat Genet 42 (2010) 448-53.

[16] D.S. Cannon, T.B. Baker, M.E. Piper, M.B. Scholand, D.L. Lawrence, D.T. Drayna, W.M. McMahon, G.M. Villegas, T.C. Caton, H. Coon, and M.F. Leppert, Associations between phenylthiocarbamide gene polymorphisms and cigarette smoking. Nicotine Tob Res 7 (2005) 853-8.

[17] J.E. Mangold, T.J. Payne, J.Z. Ma, G. Chen, and M.D. Li, Bitter taste receptor gene polymorphisms are an important factor in the development of nicotine dependence in African Americans. Journal of medical genetics 45 (2008) 578-82.

[18] J.P. Rice, S.M. Hartz, A. Agrawal, L. Almasy, S. Bennett, N. Breslau, K.K. Bucholz, K.F. Doheny, H.J. Edenberg, A.M. Goate, V. Hesselbrock, W.B. Howells, E.O. Johnson, J. Kramer, R.F. Krueger, S. Kuperman, C. Laurie, T.A. Manolio, R.J. Neuman, J.I. Nurnberger, B. Porjesz, E. Pugh, E.M. Ramos, N. Saccone, S. Saccone, M. Schuckit, and L.J. Bierut, CHRNB3 is more strongly associated with Fagerstrom test for cigarette dependence-based nicotine dependence than cigarettes per day: phenotype definition changes genome-wide association studies results. Addiction 107 (2012) 2019-28.

[19] A. Bar-Shira, M. Gana-Weisz, Z. Gan-Or, E. Giladi, N. Giladi, and A. Orr-Urtreger, CHRNB3 c.-57A>G functional promoter change affects Parkinson's disease and smoking. Neurobiology of aging 35 (2014) 2179 e1-6.

[20] N.R. Hoft, R.P. Corley, M.B. McQueen, I.R. Schlaepfer, D. Huizinga, and M.A. Ehringer, Genetic association of the CHRNA6 and CHRNB3 genes with tobacco dependence in a nationally representative sample. Neuropsychopharmacology 34 (2009) 698-706.

[21] C.T. Lee, B.F. Fuemmeler, F.J. McClernon, A. Ashley-Koch, and S.H. Kollins, Nicotinic receptor gene variants interact with attention deficient hyperactive disorder symptoms to predict smoking trajectories from early adolescence to adulthood. Addictive behaviors 38 (2013) 2683-9.

[22] N.L. Saccone, S.F. Saccone, A.L. Hinrichs, J.A. Stitzel, W. Duan, M.L. Pergadia, A. Agrawal, N. Breslau, R.A. Grucza, D. Hatsukami, E.O. Johnson, P.A. Madden, G.E. Swan, J.C. Wang, A.M. Goate, J.P. Rice, and L.J. Bierut, Multiple distinct risk loci for nicotine dependence identified by dense coverage of the complete family of nicotinic receptor subunit (CHRN) genes. Am J Med Genet B Neuropsychiatr Genet 150B (2009) 453-66.

[23] L.S. Chen, T.B. Baker, R. Grucza, J.C. Wang, E.O. Johnson, N. Breslau, D. Hatsukami, S.S. Smith, N. Saccone, S. Saccone, J.P. Rice, A.M. Goate, and L.J. Bierut, Dissection of the phenotypic and genotypic associations with nicotinic dependence. Nicotine Tob Res 14 (2012) 425-33.

[24] J.S. Zeiger, B.C. Haberstick, I. Schlaepfer, A.C. Collins, R.P. Corley, T.J. Crowley, J.K. Hewitt, C.J. Hopfer, J. Lessem, M.B. McQueen, S.H. Rhee, and M.A. Ehringer, The neuronal nicotinic receptor subunit genes (CHRNA6 and CHRNB3) are associated with subjective responses to tobacco. Hum Mol Genet 17 (2008) 724-34.

[25] R.C. Culverhouse, E.O. Johnson, N. Breslau, D.K. Hatsukami, B. Sadler, A.I. Brooks, V.M. Hesselbrock, M.A. Schuckit, J.A. Tischfield, A.M. Goate, N.L. Saccone, and L.J. Bierut, Multiple distinct CHRNB3-CHRNA6 variants are genetic risk factors for nicotine dependence in African Americans and European Americans. Addiction 109 (2014) 814-22.

[26] W.Y. Cui, S. Wang, J. Yang, S.G. Yi, D. Yoon, Y.J. Kim, T.J. Payne, J.Z. Ma, T. Park, and M.D. Li, Significant association of CHRNB3 variants with nicotine dependence in multiple ethnic populations. Mol Psychiatry 18 (2013) 1149-51.

[27] TAG, Genome-wide meta-analyses identify multiple loci associated with smoking behavior. Nat Genet 42 (2010) 441-7.

[28] A.M. Leventhal, W. Lee, A.W. Bergen, G.E. Swan, R.F. Tyndale, C. Lerman, and D.V. Conti, Nicotine dependence as a moderator of genetic influences on smoking cessation treatment outcome. Drug Alcohol Depend 138 (2014) 109-17.

[29] E. Ella, N. Sato, D. Nishizawa, S. Kageyama, H. Yamada, N. Kurabe, K. Ishino, H. Tao, F. Tanioka, A. Nozawa, C. Renyin, K. Shinmura, K. Ikeda, and H. Sugimura, Association between dopamine beta hydroxylase rs5320 polymorphism and smoking behaviour in elderly Japanese. J Hum Genet 57 (2012) 385-90.

[30] J. Beuten, J.Z. Ma, T.J. Payne, R.T. Dupont, K.M. Crews, G. Somes, N.J. Williams, R.C. Elston, and M.D. Li, Single- and multilocus allelic variants within the GABA(B) receptor subunit 2 (GABAB2) gene are significantly associated with nicotine dependence. Am J Hum Genet 76 (2005) 859-64.

[31] J.Z. Ma, T.J. Payne, and M.D. Li, Significant association of glutamate receptor, ionotropic N-methyl-D-aspartate 3A (GRIN3A), with nicotine dependence in European- and African-American smokers. Hum Genet 127 (2010) 503-12.

[32] J.M. Vink, A.B. Smit, E.J. de Geus, P. Sullivan, G. Willemsen, J.J. Hottenga, J.H. Smit, W.J. Hoogendijk, F.G. Zitman, L. Peltonen, J. Kaprio, N.L. Pedersen, P.K. Magnusson, T.D. Spector, K.O. Kyvik, K.I. Morley, A.C. Heath, N.G. Martin, R.G. Westendorp, P.E. Slagboom, H. Tiemeier, A. Hofman, A.G. Uitterlinden, Y.S. Aulchenko, N. Amin, C. van Duijn, B.W. Penninx, and D.I. Boomsma, Genome-wide association study of smoking initiation and current smoking. Am J Hum Genet 84 (2009) 367-79.

[33] J. Beuten, J.Z. Ma, T.J. Payne, R.T. Dupont, X.Y. Lou, K.M. Crews, R.C. Elston, and M.D. Li, Association of specific haplotypes of neurotrophic tyrosine kinase receptor 2 gene (NTRK2) with vulnerability to nicotine dependence in African-Americans and European-Americans. Biol Psychiatry 61 (2007) 48-55.

[34] M.D. Li, D. Sun, X.Y. Lou, J. Beuten, T.J. Payne, and J.Z. Ma, Linkage and association studies in African- and Caucasian-American populations demonstrate that SHC3 is a novel susceptibility locus for nicotine dependence. Mol Psychiatry 12 (2007) 462-73.

[35] Q. Xu, W. Huang, T.J. Payne, J.Z. Ma, and M.D. Li, Detection of genetic association and a functional polymorphism of dynamin 1 gene with nicotine dependence in European and African Americans. Neuropsychopharmacology 34 (2009) 1351-9.

[36] J. Wei, J.Z. Ma, T.J. Payne, W. Cui, R. Ray, N. Mitra, C. Lerman, and M.D. Li, Replication and extension of association of choline acetyltransferase with nicotine dependence in European and African American smokers. Hum Genet 127 (2010) 691-8.

[37] L. Zhang, K.S. Kendler, and X. Chen, Association of the phosphatase and tensin homolog gene (PTEN) with smoking initiation and nicotine dependence. Am J Med Genet B Neuropsychiatr Genet 141B (2006) 10-4.

[38] J.R. Turner, R. Ray, B. Lee, L. Everett, J. Xiang, C. Jepson, K.H. Kaestner, C. Lerman, and J.A. Blendy, Evidence from mouse and man for a role of neuregulin 3 in nicotine dependence. Mol Psychiatry 19 (2014) 801-10.

[39] S.P. David, M.R. Munafo, M.F. Murphy, M. Proctor, R.T. Walton, and E.C. Johnstone, Genetic variation in the dopamine D4 receptor (DRD4) gene and smoking cessation: follow-up of a randomised clinical trial of transdermal nicotine patch. Pharmacogenomics J 8 (2008) 122-8.

[40] J.A. Ellis, C.A. Olsson, E. Moore, P. Greenwood, M.O. Van De Ven, and G.C. Patton, A role for the DRD4 exon III VNTR in modifying the association between nicotine dependence and neuroticism. Nicotine Tob Res 13 (2011) 64-9.

[41] D. Das, X. Tan, and S. Easteal, Effect of model choice in genetic association studies: DRD4 exon III VNTR and cigarette use in young adults. Am J Med Genet B Neuropsychiatr Genet 156B (2011) 346-51.

[42] S.P. David, B. Mezuk, P.P. Zandi, D. Strong, J.C. Anthony, R. Niaura, G.R. Uhl, and W.W. Eaton, Sex differences in TTC12/ANKK1 haplotype associations with daily tobacco smoking in Black and White Americans. Nicotine Tob Res 12 (2010) 251-62.

[43] P. Yudkin, M. Munafo, K. Hey, S. Roberts, S. Welch, E. Johnstone, M. Murphy, S. Griffiths, and R. Walton, Effectiveness of nicotine patches in relation to genotype in women versus men: randomised controlled trial. BMJ 328 (2004) 989-90.

[44] E.C. Johnstone, P.L. Yudkin, K. Hey, S.J. Roberts, S.J. Welch, M.F. Murphy, S.E. Griffiths, and R.T. Walton, Genetic variation in dopaminergic pathways and short-term effectiveness of the nicotine patch. Pharmacogenetics 14 (2004) 83-90.

[45] C. Lerman, C. Jepson, E.P. Wileyto, L.H. Epstein, M. Rukstalis, F. Patterson, V. Kaufmann, S. Restine, L. Hawk, R. Niaura, and W. Berrettini, Role of functional genetic variation in the dopamine D2 receptor (DRD2) in response to bupropion and nicotine replacement therapy for tobacco dependence: results of two randomized clinical trials. Neuropsychopharmacology 31 (2006) 231-42.

[46] D.E. Comings, L. Ferry, S. Bradshaw-Robinson, R. Burchette, C. Chiu, and D. Muhleman, The dopamine D2 receptor (DRD2) gene: a genetic risk factor in smoking. Pharmacogenetics 6 (1996) 73-9.

[47] J. Gelernter, Y. Yu, R. Weiss, K. Brady, C. Panhuysen, B.Z. Yang, H.R. Kranzler, and L. Farrer, Haplotype spanning TTC12 and ANKK1, flanked by the DRD2 and NCAM1 loci, is strongly associated to nicotine dependence in two distinct American populations. Hum Mol Genet 15 (2006) 3498-507.

[48] L.C. Bidwell, M.E. Garrett, F.J. McClernon, B.F. Fuemmeler, R.B. Williams, A.E. Ashley-Koch, and S.H. Kollins, A preliminary analysis of interactions between genotype, retrospective ADHD symptoms, and initial reactions to smoking in a sample of young adults. Nicotine Tob Res 14 (2012) 229-33.

[49] W. Huang, T.J. Payne, J.Z. Ma, J. Beuten, R.T. Dupont, N. Inohara, and M.D. Li, Significant association of ANKK1 and detection of a functional polymorphism with nicotine dependence in an African-American sample. Neuropsychopharmacology 34 (2009) 319-30.

[50] F. Ducci, M. Kaakinen, A. Pouta, A.L. Hartikainen, J. Veijola, M. Isohanni, P. Charoen, L. Coin, C. Hoggart, J. Ekelund, L. Peltonen, N. Freimer, P. Elliott, G. Schumann, and M.R. Jarvelin, TTC12-ANKK1-DRD2 and CHRNA5-CHRNA3-CHRNB4 influence different pathways leading to smoking behavior from adolescence to mid-adulthood. Biol Psychiatry 69 (2011) 650-60.

[51] J. Gelernter, C. Panhuysen, R. Weiss, K. Brady, J. Poling, M. Krauthammer, L. Farrer, and H.R. Kranzler, Genomewide linkage scan for nicotine dependence: identification of a chromosome 5 risk locus. Biol Psychiatry 61 (2007) 119-26.

[52] Z. Yang, C. Seneviratne, S. Wang, J.Z. Ma, T.J. Payne, J. Wang, and M.D. Li, Serotonin transporter and receptor genes significantly impact nicotine dependence through genetic interactions in both European American and African American smokers. Drug Alcohol Depend 129 (2013) 217-25.

[53] X.Y. Lou, J.Z. Ma, T.J. Payne, J. Beuten, K.M. Crew, and M.D. Li, Gene-based analysis suggests association of the nicotinic acetylcholine receptor beta1 subunit (CHRNB1) and M1 muscarinic acetylcholine receptor (CHRM1) with vulnerability for nicotine dependence. Hum Genet 120 (2006) 381-9.

[54] X.Y. Zhang, C. Chen da, M.H. Xiu, X. Luo, L. Zuo, C.N. Haile, T.A. Kosten, and T.R. Kosten, BDNF Val66Met variant and smoking in a Chinese population. PLoS One 7 (2012) e53295.

[55] J. Beuten, J.Z. Ma, T.J. Payne, R.T. Dupont, P. Quezada, W. Huang, K.M. Crews, and M.D. Li, Significant association of BDNF haplotypes in European-American male smokers but not in European-American female or African-American smokers. Am J Med Genet B Neuropsychiatr Genet 139B (2005) 73-80.

[56] D. Sun, J.Z. Ma, T.J. Payne, and M.D. Li, Beta-arrestins 1 and 2 are associated with nicotine dependence in European American smokers. Mol Psychiatry 13 (2008) 398-406.

[57] G.B. Chen, T.J. Payne, X.Y. Lou, J.Z. Ma, J. Zhu, and M.D. Li, Association of amyloid precursor protein-binding protein, family B, member 1 with nicotine dependence in African and European American smokers. Hum Genet 124 (2008) 393-8.

[58] M.D. Li, J.Z. Ma, T.J. Payne, X.Y. Lou, D. Zhang, R.T. Dupont, and R.C. Elston, Genome-wide linkage scan for nicotine dependence in European Americans and its converging results with African Americans in the Mid-South Tobacco Family sample. Mol Psychiatry 13 (2008) 407-16.

[59] J. Beuten, J.Z. Ma, X.Y. Lou, T.J. Payne, and M.D. Li, Association analysis of the protein phosphatase 1 regulatory subunit 1B (PPP1R1B) gene with nicotine dependence in European- and African-American smokers. Am J Med Genet B Neuropsychiatr Genet 144B (2007) 285-90.

[60] X.Y. Lou, J.Z. Ma, D. Sun, T.J. Payne, and M.D. Li, Fine mapping of a linkage region on chromosome 17p13 reveals that GABARAP and DLG4 are associated with vulnerability to nicotine dependence in European-Americans. Hum Mol Genet 16 (2007) 142-53.

[61] I. Kremer, R. Bachner-Melman, A. Reshef, L. Broude, L. Nemanov, I. Gritsenko, U. Heresco-Levy, Y. Elizur, and R.P. Ebstein, Association of the serotonin transporter gene with smoking behavior. The American journal of psychiatry 162 (2005) 924-30.

[62] J. Daw, J.D. Boardman, R. Peterson, A. Smolen, B.C. Haberstick, M.A. Ehringer, S.T. Ennett, and V.A. Foshee, The interactive effect of neighborhood peer cigarette use and 5HTTLPR genotype on individual cigarette use. Addictive behaviors 39 (2014) 1804-10.

[63] E. Johnstone, N. Benowitz, A. Cargill, R. Jacob, L. Hinks, I. Day, M. Murphy, and R. Walton, Determinants of the rate of nicotine metabolism and effects on smoking behavior. Clin Pharmacol Ther 80 (2006) 319-30.

[64] L.S. Chen, A.J. Bloom, T.B. Baker, S.S. Smith, M.E. Piper, M. Martinez, N. Saccone, D. Hatsukami, A. Goate, and L. Bierut, Pharmacotherapy effects on smoking cessation vary with nicotine metabolism gene (CYP2A6). Addiction 109 (2014) 128-37.

[65] A.J. Bloom, T.B. Baker, L.S. Chen, N. Breslau, D. Hatsukami, L.J. Bierut, and A. Goate, Variants in two adjacent genes, EGLN2 and CYP2A6, influence smoking behavior related to disease risk via different mechanisms. Hum Mol Genet 23 (2014) 555-61.

[66] N. Kumasaka, M. Aoki, Y. Okada, A. Takahashi, K. Ozaki, T. Mushiroda, T. Hirota, M. Tamari, T. Tanaka, Y. Nakamura, N. Kamatani, and M. Kubo, Haplotypes with copy number and single nucleotide polymorphisms in CYP2A6 locus are associated with smoking quantity in a Japanese population. PLoS One 7 (2012) e44507.

[67] A. Loukola, J. Buchwald, R. Gupta, T. Palviainen, J. Hallfors, E. Tikkanen, T. Korhonen, M. Ollikainen, A.P. Sarin, S. Ripatti, T. Lehtimaki, O. Raitakari, V. Salomaa, R.J. Rose, R.F. Tyndale, and J. Kaprio, A Genome-Wide Association Study of a Biomarker of Nicotine Metabolism. PLoS Genet 11 (2015) e1005498.

[68] Y. Feng, T.H. Niu, H.X. Xing, X. Xu, C.Z. Chen, S.J. Peng, L.H. Wang, N. Laird, and X.P. Xu, A common haplotype of the nicotine acetylcholine receptor alpha 4 subunit gene is associated with vulnerability to nicotine addiction in men. American Journal of Human Genetics 75 (2004) 112-121.

[69] M.D. Li, J. Beuten, J.Z. Ma, T.J. Payne, X.Y. Lou, V. Garcia, A.S. Duenes, K.M. Crews, and R.C. Elston, Ethnic- and gender-specific association of the nicotinic acetylcholine receptor alpha4 subunit gene (CHRNA4) with nicotine dependence. Hum Mol Genet 14 (2005) 1211-9.

[70] H.M. Kamens, R.P. Corley, M.B. McQueen, M.C. Stallings, C.J. Hopfer, T.J. Crowley, S.A. Brown, J.K. Hewitt, and M.A. Ehringer, Nominal association with CHRNA4 variants and nicotine dependence. Genes Brain Behav 12 (2013) 297-304.

[71] L.P. Breitling, N. Dahmen, K. Mittelstrass, D. Rujescu, J. Gallinat, C. Fehr, I. Giegling, C. Lamina, T. Illig, H. Muller, E. Raum, D. Rothenbacher, H.E. Wichmann, H. Brenner, and G. Winterer, Association of nicotinic acetylcholine receptor subunit alpha 4 polymorphisms with nicotine dependence in 5500 Germans. Pharmacogenomics J 9 (2009) 219-24.

[72] P. Xie, H.R. Kranzler, M. Krauthammer, K.P. Cosgrove, D. Oslin, R.F. Anton, L.A. Farrer, M.R. Picciotto, J.H. Krystal, H. Zhao, and J. Gelernter, Rare nonsynonymous variants in alpha-4 nicotinic acetylcholine receptor gene protect against nicotine dependence. Biol Psychiatry 70 (2011) 528-36.

[73] W.H. Berrettini, E.P. Wileyto, L. Epstein, S. Restine, L. Hawk, P. Shields, R. Niaura, and C. Lerman, Catechol-O-methyltransferase (COMT) gene variants predict response to bupropion therapy for tobacco dependence. Biol Psychiatry 61 (2007) 111-8.

[74] A.B. Amstadter, N.R. Nugent, K.C. Koenen, K.J. Ruggiero, R. Acierno, S. Galea, D.G. Kilpatrick, and J. Gelernter, Association between COMT, PTSD, and increased smoking following hurricane exposure in an epidemiologic sample. Psychiatry 72 (2009) 360-9.

[75] G. Nedic, M. Nikolac, F. Borovecki, S. Hajnsek, D. Muck-Seler, and N. Pivac, Association study of a functional catechol-O-methyltransferase polymorphism and smoking in healthy Caucasian subjects. Neuroscience letters 473 (2010) 216-9.

[76] J. Beuten, T.J. Payne, J.Z. Ma, and M.D. Li, Significant association of catechol-O-methyltransferase (COMT) haplotypes with nicotine dependence in male and female smokers of two ethnic populations. Neuropsychopharmacology 31 (2006) 675-84.

[77] M. Omidvar, L. Stolk, A.G. Uitterlinden, A. Hofman, C.M. Van Duijn, and H. Tiemeier, The effect of catechol-O-methyltransferase Met/Val functional polymorphism on smoking cessation: retrospective and prospective analyses in a cohort study. Pharmacogenet Genomics 19 (2009) 45-51.

[78] M.R. Munafo, R.M. Freathy, S.M. Ring, B. St Pourcain, and G.D. Smith, Association of COMT Val(108/158)Met genotype and cigarette smoking in pregnant women. Nicotine Tob Res 13 (2011) 55-63.

[79] J. Yang, and M.D. Li, Converging findings from linkage and association analyses on susceptibility genes for smoking and other addictions. Mol Psychiatr (2016).
