# Supplementary material for: Investigation of the genetic effect of 56 tobacco-smoking susceptibility genes on DNA methylation and RNA expression in human brain
Source: Front Psychiatry. 2022 Aug 18;13:924062. doi: 10.3389/fpsyt.2022.924062 (PMC9433921; doi:10.3389/fpsyt.2022.924062)
Supplement: Supplementary Figure 1 — Linkage disequilibrium (LD) D’ plot of the 54 eQTLs for EGLN2 gene in BrainCloud EA sample. The LD D’ plot was drawn using Haploview (https://www.broadinstitute.org/scientific-community/science/programs/medical-and-population-genetics/haploview/haploview). Gene annotation and regulatory feature tracks were obtained from the 1000 Genomes Browser (http://www.internationalgenome.org/1000-genomes-browers/index.html). The black vertical bar indicates position of expression probe hHC023008. Red boxes highlight eight rare variants, which were found to be collectively affecting smoking quantity by Clark et al. (32). The yellow star marks the variant with the strongest biological evidence based on HaploReg v4.1 results (http://www.broadinstitute.org/mammals/haploreg/haploreg.php). [file Presentation_1.PPTX]

## Slide 1
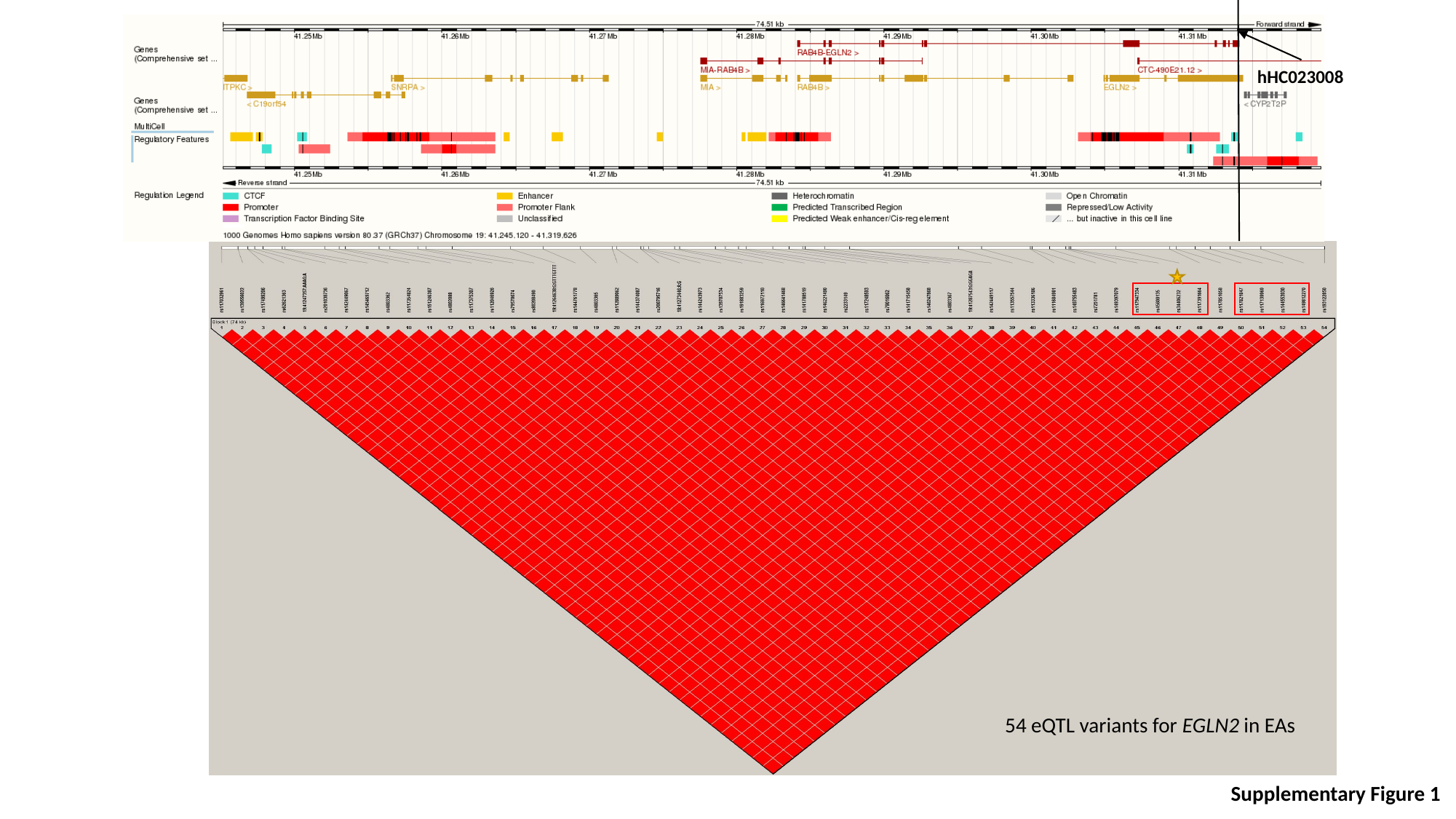

hHC023008
54 eQTL variants for EGLN2 in EAs
Supplementary Figure 1

## Slide 2
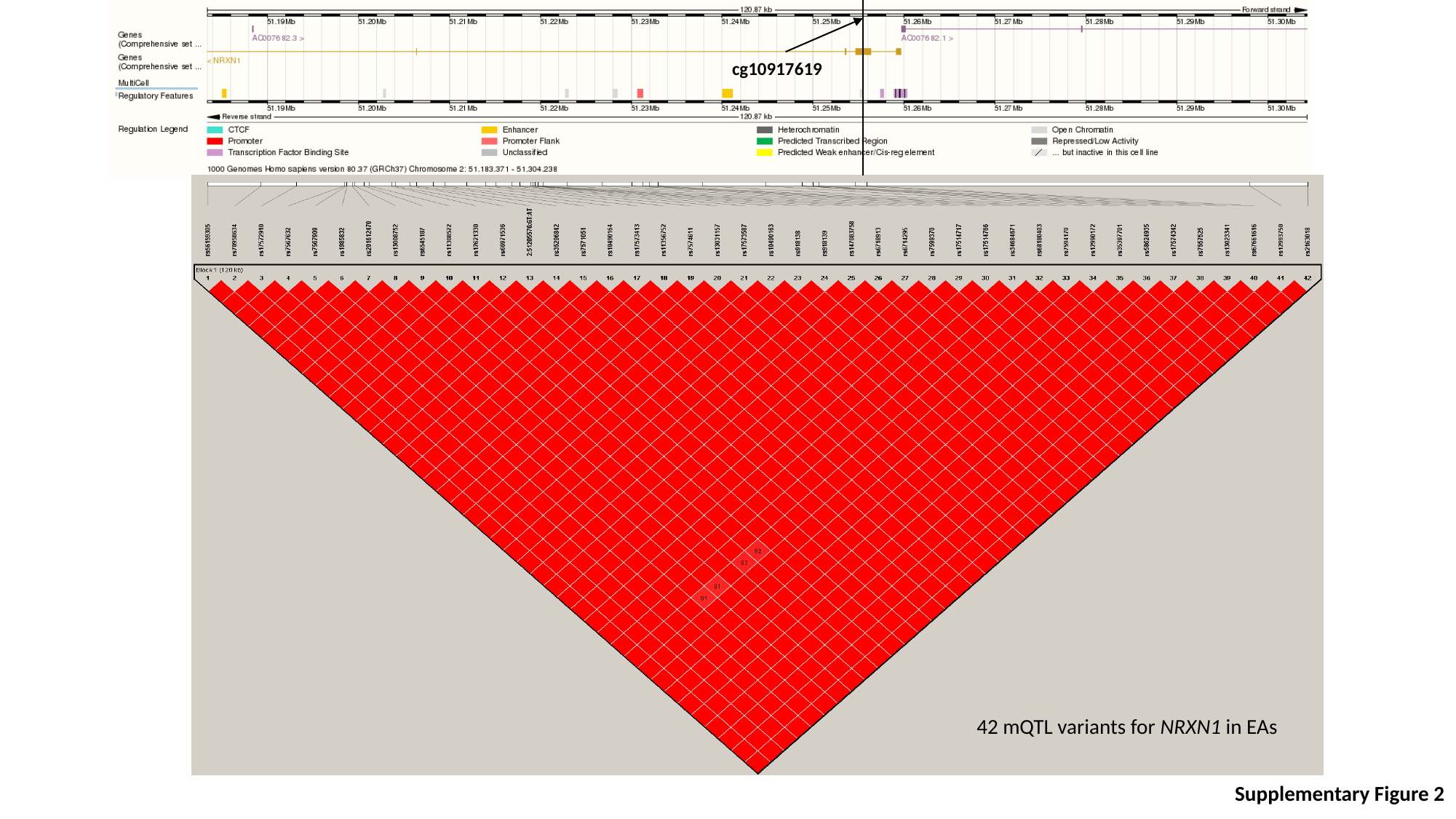

42 mQTL variants for NRXN1 in EAs
cg10917619
Supplementary Figure 2

## Slide 3
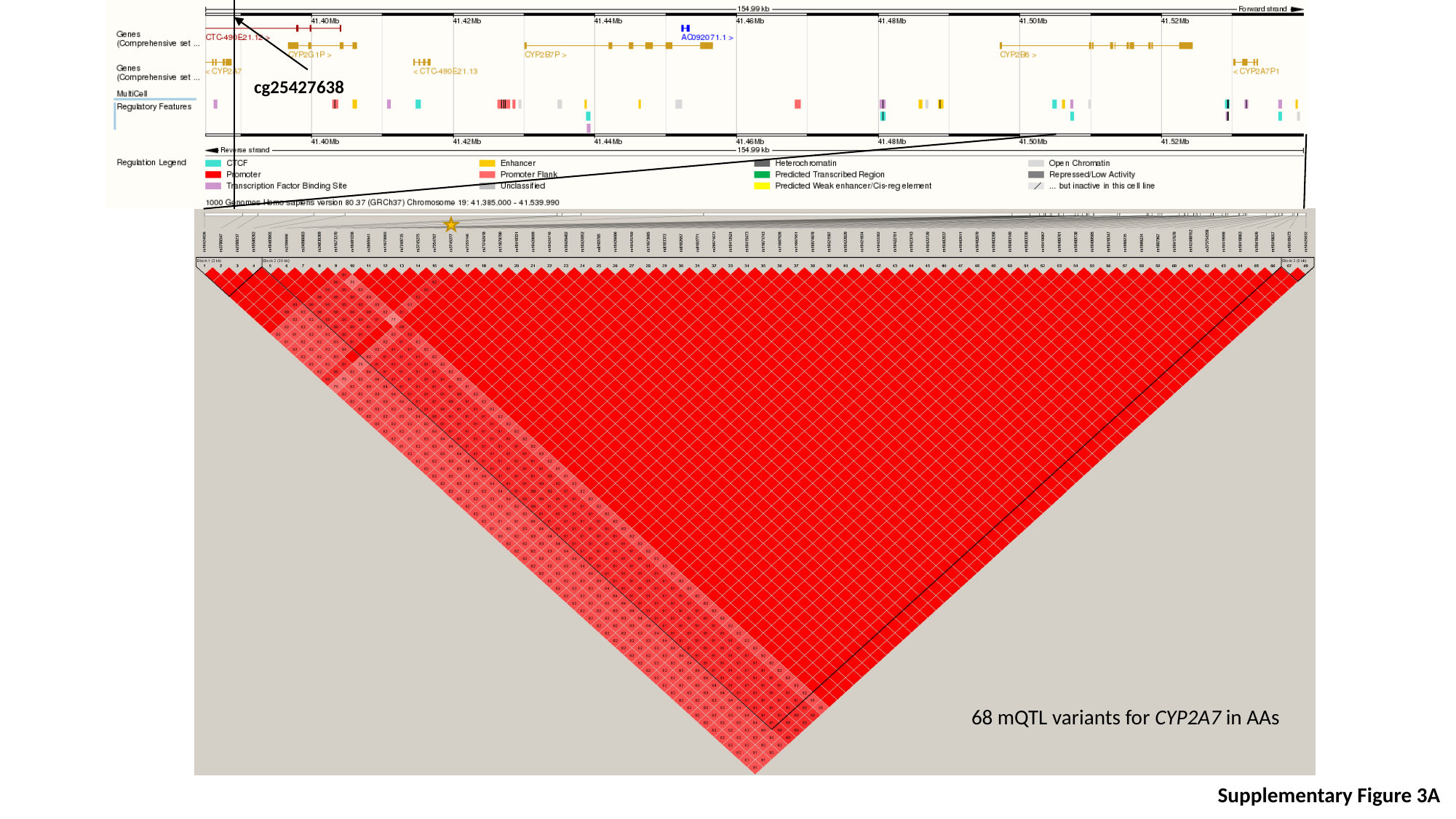

68 mQTL variants for CYP2A7 in AAs
cg25427638
Supplementary Figure 3A

## Slide 4
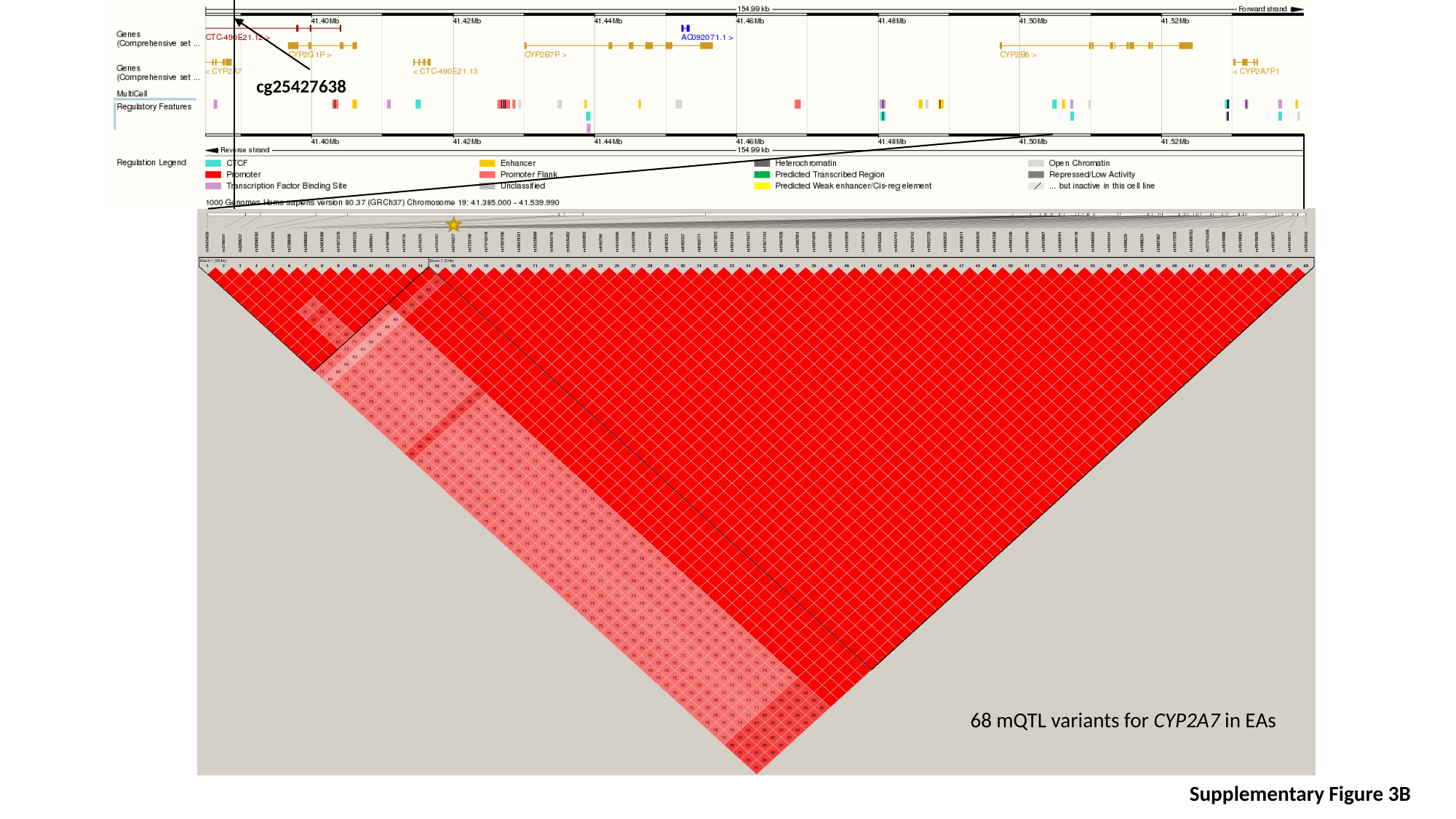

cg25427638
68 mQTL variants for CYP2A7 in EAs
Supplementary Figure 3B
